# A Factor‐Analytic Approach for Psychometric Properties of the Repetitive Behaviors Questionnaire‐3 (RBQ‐3) for Turkish Population

**DOI:** 10.1002/aur.70311

**Published:** 2026-07-15

**Authors:** Volkan Avşar, Hatice Şengül Erdem, Asiye Şengül Avşar

**Affiliations:** ^1^ Faculty of Education, Department of Psychological Counselling and Guidance in Educational Sciences Recep Tayyip Erdoğan University Rize Türkiye; ^2^ Faculty of Education Sciences, Department of Special Education Istanbul Medeniyet University Istanbul Türkiye; ^3^ Faculty of Education, Department of Measurement and Evaluation in Educational Sciences Recep Tayyip Erdoğan University Rize Türkiye

**Keywords:** autism spectrum disorders, factor‐analytic methods, restricted and repetitive behaviors, scale adaptation

## Abstract

Restricted and repetitive behaviors (RRBs) are one of the diagnostic criteria for autism spectrum disorders (ASD), as well as being found in the general population. The current study aims to explore the psychometric properties of the Turkish self‐report and informant‐report forms of the Repetitive Behaviors Questionnaire‐3 (RBQ‐3) for individuals with ASD and the general population across the lifespan. A series of factor‐analytic methods was employed to analyze data from study groups with diverse characteristics. A total of 1803 individuals participated in the study. Bayesian confirmatory analysis, confirmatory factor analysis, exploratory structural equation modeling, the average variance extracted, and measurement invariance analyses were performed to provide evidence of construct validity for the Turkish RBQ‐3's in both self—and informant forms. In addition, the reliability of the Turkish RBQ‐3 scores was evaluated using Cronbach's alpha, McDonald's omega, and composite reliability. In conclusion, the adapted self‐report and informant‐report forms of the RBQ‐3 demonstrate strong psychometric properties for assessing RRBs in both individuals with ASD and the wider population across the lifespan in Türkiye. The Turkish RBQ‐3 offers a valuable tool for Turkish researchers and clinicians, advancing the understanding of RRBs.

## 1 Introduction

Although restricted and repetitive behaviors (RRBs) are one of the diagnostic criteria for autism spectrum disorders (ASD) (American Psychiatric Association 2013), there is a wide range of presentation of RRBs across genetic and clinical conditions (e.g., Prader‐Willi syndrome, Down syndrome, obsessive‐compulsive disorder, attention deficit hyperactivity disorder) (Keating et al. 2023) as well as in the general population (Evans et al. 2017). The Diagnostic and Statistical Manual of Mental Disorders (DSM‐5) defines RRBs as repetitive motor movements, insistence on sameness, restricted interests, and unusual sensitivity to sensory input (American Psychiatric Association 2013). More specifically, RRBs are heterogeneous wide set of behaviors including highly repetitive motor behaviors such as flapping hands; inflexible use of objects such as spinning of objects, unusual sensory features, rigid adherence to rituals such as insisting that items are arranged on the dinner table in a certain way, intense interest with objects such as leaves on trees, and restricted activities that resist change and are driven by a desire for sameness (Barrett et al. 2015; Jones et al. 2024; Leekam et al. 2011; Richler et al. 2007; Uljarević et al. 2023).

Despite the different categorization of RRBs, such as lower order‐higher order RRBs (Turner 1999), repetitive sensory motor (RMS), insistence on sameness (IS) (Richler et al. 2010), self‐injurious behavior, stereotyped behaviors, restricted behavior, compulsive behavior, ritualistic/sameness behavior (Lam and Aman 2007), it is clear that RRBs need further understanding for many purposes. Firstly, and perhaps most importantly, since RRBs are fundamental features of ASD, a precise understanding of these behaviors from early childhood to adulthood is critical for a comprehensive understanding of ASD (Richler et al. 2007). Since the beginning of the first year (e.g., 2 and 3), the presence of RRBs might be indicative of the identification of ASD (Arnott et al. 2010; Chaxiong et al. 2022; Wolff et al. 2014), and they are meaningful predictors of well‐being‐emotional and behavioral difficulties, poorer language and cognitive characteristics from an early age (e.g., at age 6) (Carrington et al. 2023), the assessment of RRBs should cover the early age period (Richler et al. 2007).

RRBs can pose significant challenges for some individuals with ASD and their families; these challenges may include considerable family distress and dysfunction, often arising from intolerance of change and behaviors such as aggression toward oneself or others (Bishop et al. 2013; Zandt et al. 2007). In such cases, as a core characteristic of ASD, RRBs should be included in the intervention, rigorously measured for diagnosis, and clearly defined (Leekam et al. 2011). A rigorous assessment requires a tool that covers a wide range of RRBs, captures their type and intensity, and indicates how frequently they are experienced (Jones et al. 2024). This type of assessment might lead clinicians or educators to effective interventions, as well as researchers conducting longitudinal studies.

RRBs are not unique to childhood or the early ages. RRBs are heterogeneous and may exhibit distinct patterns across the lifespan, depending on age‐related profiles (Esbensen et al. 2009; Kumar et al. 2022). While Esbensen et al. (2009) indicated that RRBs tend to be less frequent and less severe in older individuals with ASD than in younger individuals, Kumar et al. (2022) reported that older children, aged 3–8, with more severe ASD characteristics had significantly higher levels of RRBs. These inconsistent results highlight the need to better understand age‐related changes in RRBs, which may contribute to practitioners' more comprehensive understanding of ASD (Chowdhury et al. 2010; Esbensen et al. 2009). Additionally, many behavioral characteristics of ASD that require intervention may be related to RRBs. For example, Duerden et al. (2012) found that atypical sensory processing and insistence on sameness were significant predictors of self‐injurious behaviors in children and adolescents with ASD. In a longitudinal study, Laverty et al. (2020) investigated self‐injurious behaviors in individuals with ASD over 10 years and found that 44% were persistent and were positively associated with repetitive behaviors. The authors recommended using a standardized screening tool to support the clinical implications of these conclusions.

RRBs are also associated with depression and anxiety in individuals with ASD (Muskett et al. 2019; Rodgers et al. 2012). The severity of RRBs is correlated with more anxiety (Lidstone et al. 2014; Sellick et al. 2021) and significant indicators of anxiety in the future in children with ASD (Baribeau et al. 2020). Additionally, RRBs co‐occur with mental health problems (externalizing and internalizing behaviors) in the ASD population (Jasim and Perry 2023). In such cases, understanding the relation between RRBs and these constructs is significant for effective intervention.

Some individuals with ASD may have less insight into their own behaviors compared to individuals without ASD (DeBrabander et al. 2021). On the other hand, it is proposed that novel self‐report instruments are needed to better capture personal insights into ASD traits, thereby facilitating a deeper understanding for researchers and practitioners beyond traditional diagnostic methods (Ratto et al. 2023). As a core characteristic of ASD, a combined assessment of RRBs using both self‐report and informant‐report tools may enhance assessment accuracy. At this point, measurement tools that provide valid and reliable results in measuring RBBs are noteworthy.

The Repetitive Behaviors Questionnaire‐3 (RBQ‐3) was developed by Jones et al. (2024) as a 20‐item lifetime questionnaire. It revises the RBQ‐parent questionnaire for children's RRBs (RBQ‐2) and RBQ‐2 for adults (RBQ‐2A). It has two forms: self‐report and informant‐report for use across the lifespan (Jones et al. 2024). Jones et al. (2024) conducted the first psychometric study of both forms. This study, with adults without intellectual disability, found the questionnaire to be valid and reliable for assessing RRBs across the lifespan in clinical and research settings. The authors also recommended further research with other groups, such as children and adults with ASD and their family members.

The RBQ‐3 self‐report (RBQ‐3‐S) is a scale that assesses RRBs in individuals (Jones et al. 2024). No direct study has demonstrated the construct validity of the RBQ‐3‐S using factor‐analytic methods. Jones et al. (2024) conducted a psychometric evaluation of the RBQ‐3. The study used the self‐report and informant‐report forms of the RBQ‐3. In the Jones et al. (2024) study, individuals with ASD responded to self‐report, and their parents, spouses, other family members, or long‐term friends responded to informant‐report. A high correlation was found between the scores obtained from the forms. The RBQ‐3‐S comprises the same items as the RBQ‐2A; however, their scoring methods differ. All items included in the scoring (the first 19 items) are scored on a four‐point ordinal Likert‐type scale in the RBQ‐3‐S. However, all items in the RBQ‐2A are scored on a three‐point ordinal Likert‐type scale (Barrett et al. 2015). The factor structures and item content of the RBQ‐2A reported across studies are shown in Table 1.

**TABLE 1:** Studies revealing the factor structure of the RBQ‐2A.

Authors	Sample characteristics	Method	Findings	Items excluded
Barrett et al. (2015)	Neurotypical adults and university students	Principal components analysis (PCA)	Repetitive motor behavior (1, 2, 3, 4, 5, 6) Insistence on sameness (11, 12, 13, 14, 15, 16, 17, 19)	7, 8, 9, 10 and 18
Barrett et al. (2018)	Adults with ASD	Polychoric PCA with Direct oblimin rotation	Repetitive sensory motor behavior (2, 3, 4, 5, 6, 10) Insistence on sameness (1, 7, 11, 12, 13, 14, 15, 16, 17, 18, 19)	8 and 9
Brett et al. (2024)	University students	ESEM and CFA (Modified four‐factor model)	Repetitive motor behaviors (2, 3, 4, 5, 6) Interest in sensations & objects (7, 8, 10, 11, 12) Insistence on sameness (13, 14, 15, 16) Restricted interests (17, 18, 19, 20)	1 and 9
Brett et al. (2024)	Adults with ASD	CFA—hierarchical model	Repetitive motor behaviors (2, 3, 4, 5, 6) Interest in sensations & Objects (7, 8, 10, 11, 12) Insistence on sameness (13, 14, 15, 16) Restricted interests (17, 18, 19, 20)	1 and 9

The RBQ‐2A generally consists of either a two‐factor or a four‐factor structure, with differing item compositions across adults with ASD and neurotypical adults, as presented in Table 1. In the study by Jones et al. (2024) examining the psychometric properties of the RBQ‐3‐S, the two‐factor structure identified by Barrett et al. (2018) based on data from adults with ASD was adopted. This two‐factor structure was used as the basis for the RBQ‐3‐S.

The RBQ‐3 informant‐report (RBQ‐3‐I) is a scale that assesses RRBs in individuals (Jones et al. 2024). The RBQ‐3‐I should be filled out by a relative, caregiver, or someone closely acquainted with the individual. It is suitable for documenting RRBs in children and adults of all ages. No study has demonstrated the construct validity of the RBQ‐3‐I using factor‐analytic methods. As previously mentioned, Jones et al. (2024) conducted a psychometric evaluation of the RBQ‐3 using non‐factor analytic methods. The RBQ‐3‐I contains the same items as the RBQ‐2, although the scoring methods differ between the two scales. All items included in scoring (the first 19 items) are on a four‐point ordinal Likert‐type scale in the RBQ‐3‐I. In contrast, the scoring in the RBQ‐2 is heterogeneous: items 1 to 6 and 13 to 19 are scored on a four‐point ordinal Likert‐type scale, while items 7 to 12 are scored on a three‐point ordinal Likert‐type scale (Barrett et al. 2015). The factor structures of the RBQ‐2 revealed in various studies are shown in Table 2.

**TABLE 2:** Studies revealing the factor structure of the RBQ‐2.

Authors	Sample characteristics	Method	Findings	Items excluded
Lidstone et al. (2014)	2–17‐year‐olds individuals with ASD	PCA with varimax rotation	Repetitive motor and sensory behaviors (2, 3, 4, 5, 6, 8, 10, 19) Insistence on sameness (7, 9, 12, 13, 14, 15, 16, 17, 18)	1 and 11
Uljarević, Arnott, et al. (2017), Uljarević, Richdale, et al. 2017	6‐year‐old typically developing children	Categorical PCA with varimax rotation using Kaiser normalization	Repetitive motor and sensory behaviors: 2, 3, 4, 5, 6, 8, 10, 19 Insistence on sameness: 7, 9, 12, 13, 14, 15, 16, 17, 18	1 and 11
Leekam et al. (2007)	Typically developing 2‐year‐olds	PCA with varimax rotation	Repetitive Motor Movements (1, 2, 3, 4, 5, 6, 8, 9, 10) Rigidity/Routines/Preoccupation with restricted interests (11, 13, 14, 15, 16, 17,18, 19)	7 and 12
Leekam et al. (2007)	Typically developing 2‐year‐olds	PCA with varimax rotation	Repetitive Motor Movements (2, 3, 4, 5, 6) Rigidity/Adherence to Routine (13, 14, 15, 16, 17*, 18*, 19) Preoccupation with Restricted Patterns of Interest (1, 7, 8*, 10*, 11, 12, 17) Unusual Sensory Interest (8*, 9, 10*, 18*)	

*Items 8, 10, 17, and 18 are associated with two factors.

The RBQ‐2 is administered to children with ASD and neurotypical children, as shown in Table 2. In addition, it is clear that a two‐factor structure is generally observed in studies; however, a four‐factor structure is also reached. It is also seen that some items were omitted. In the study by Jones et al. (2024) examining the psychometric properties of the RBQ‐3‐I, the RBQ‐2A structure identified by Barrett et al. (2018) with data obtained from adults with ASD was taken into account.

Overall, the RBQ‐3 is the revised version of the RBQ‐2 and the RBQ‐2A (Jones et al. 2024). Both are widely used and psychometrically evaluated tools in the literature. Psychometric studies on the newly developed RBQ‐3 are limited. The only existing study was conducted by Jones et al. (2024). It is important that the RBQ‐3 provides a comprehensive assessment of RRBs, functions as a brief measure that can be completed by multiple informants, including self‐report, parents, teachers, and clinicians, and demonstrates utility in supporting autism diagnostic services (Jones et al. 2024). Therefore, our study aimed to adapt the Turkish form of the RBQ‐3 across different study groups using multiple‐factor analysis techniques.

Given the rising prevalence of ASD, capturing one of the core characteristics with a brief and different‐informant measure becomes remarkable for diagnosis (Jones et al. 2024). Beyond that, RRBs are also found in other clinical and non‐clinical populations (Keating et al. 2023; Leekam et al. 2007). Understanding the differential presence of RRBs across typical development, ASD, and other clinical populations (e.g., Down syndrome) may provide valuable insights into the mechanisms underlying the atypical manifestation of RRBs in infants, children, and adults across these groups (Berry et al. 2018).

Measuring RRBs associated with ASD is also a significant concern in Türkiye. Important studies are being conducted in Türkiye for the United Nations Sustainable Development Goals of Good Health and Well‐being, Quality Education, Reduced Inequalities, Peace, Justice and Strong Institutions, and Decent Work and Economic Growth (https://sdgresources.relx.com/autism). The 2nd National Action Plan for Individuals with Autism Spectrum Disorders 2023–2030, prepared by the Ministry of Family and Social Services, is one of the most important indicators of this. The 5th objective of this action plan includes the development and adaptation of measurement tools related to ASD (https://www.aile.tr/media/193234/otizm_eylem_plani_2023_2030.pdf).

Measurement instruments that directly assess RRBs are limited in Türkiye, and the respondents are parents or carers. Repetitive Behavior Scale‐Revised (RBS‐R) (Bodfish et al. 2000), adapted into Turkish by Ökçün‐Akçamuş et al. (2019), is the only instrument that directly assesses RRBs. While the RBS‐R, with its six‐factor structure and 43 items, has been found to be a reliable and valid tool for assessing the severity and types of RRBs in individuals with ASD, there is a need for shorter assessment tools that incorporate different informants for the Turkish population. The current study aims to explore the validity and reliability of the RBQ‐3, both the self‐report and informant‐report forms, among the Turkish neurotypical and ASD populations.

In Jones et al. (2024), the study that revealed the psychometric properties of the RBQ‐3, data from adults with ASD and their clinicians were taken into consideration. However, the factor structure of the RBQ‐3 has not been investigated using factor‐analytic methods. In our study, we first carried out the Turkish translation of the RBQ‐3 (including translation, back‐translation, and expert review). We then assessed the factor structure of the RBQ‐3 using various factor‐analytic methods. The process of determining the factor structures of the RBQ‐3‐S and the RBQ‐3‐I, examined within the scope of the current study, differs in the sampling strategies employed.

The factor structure of the RBQ‐3‐S was initially defined within a sample consisting of neurotypical individuals (Study 1), after which the validity of this structure was tested in an independent sample comprising individuals with ASD (Study 2). A different strategy was adopted for the RBQ‐3‐I, as all informants completing this scale were neurotypical individuals. Because recruiting neurotypical informants is relatively easier, the factor structure of the RBQ‐3‐I was first determined using a mixed sample of neurotypical informants who reported on both neurotypical individuals and individuals with ASD (Study 3). Subsequently, the RBQ‐3‐I's factor structure was confirmed in two independent samples: neurotypical informants reporting on individuals with ASD (Study 4) and neurotypical informants reporting on neurotypical individuals (Study 5).

## 2 Methods

This section consists of two main parts. The first part, *General Methods*, presents information including participants, measurement tools, the translation process, procedures, and the statistical analysis approach. The second part, *Study‐Specific Methods*, addresses each study individually in terms of its specific objective, participant inclusion process, participant characteristics, and the statistical analysis approach. Statistical analysis differed across the five distinct study groups depending on specific objectives, sample characteristics, and versions of RBQ‐3.

### 2.1 General Methods

This section first presents the general characteristics of the participants. It then describes the measurement instruments and the processes for adapting these instruments into Turkish. Finally, the procedures and the statistical analyses are explained in detail.

#### 2.1.1 Participants

The data were collected from 1803 participants across five independent samples. Individuals in the study samples were selected according to the purpose the scales were designed to measure, in order to test the scales' psychometric properties across different groups. For the RBQ‐3‐S, the scale was completed by neurotypical individuals and individuals with ASD themselves. For the RBQ‐3‐I, the scale was completed by informants (e.g., teachers and parents). These informants provided reports on two groups: neurotypical individuals and individuals with ASD.

#### 2.1.2 Measures

The present study used the RBQ‐3‐S and the RBQ‐3‐I. Both forms consist of the same 20 items adapted from earlier versions of the scales. The RBQ‐3‐S is suitable for use by adolescents and adults from 13 years and upwards (NovoPsych 2025). However, it is stated that older children, adolescents, and adults within the normal intelligence range can use the RBQ‐3‐S and the RBQ‐3‐I forms together, or the RBQ‐3‐S alone (Cardiff University 2025; Jones et al. 2024). The RBQ‐3‐S form should be completed by individuals for themselves.

The RBQ‐3‐I was developed for children or adults to be responded to by those who know them well, with no age limit. For individuals who are unable to complete self‐report measures, such as young children or those with significant intellectual disabilities, the RBQ‐3‐I can be administered as an independent questionnaire and completed by parents, guardians, or support staff (Jones et al. 2024). It is designed to be completed by informants who are familiar with the individual's daily behaviors and routines.

The RBQ‐3‐S is based on the RBQ‐2A (a self‐report measure), while the RBQ‐3‐I is based on the RBQ‐2 (an informant‐report measure). The only difference between the two RBQ‐3 forms lies in the phrasing of the questions: the RBQ‐3‐S asks participants about their own behaviors (e.g., “Do you like to arrange items in rows or patterns?”), whereas the RBQ‐3‐I asks informants (e.g., parents or close acquaintances) to rate the behaviors of the target individual (e.g., “Does your child, relative, or the individual you know well: Like to arrange items in rows or patterns?”). The RBQ‐3‐S and the RBQ‐3‐I differ from earlier versions of the scale in that they resolve scoring inconsistencies and are suitable for use across the life span (Jones et al. 2024).

The RBQ‐3, both forms' items 1 to 19 are scored on a Likert scale (1 to 4), and scaling is applied to these items. Item 20 can be evaluated separately. The mean total score is recommended rather than the total score to avoid missing values.

##### 2.1.2.1 Translation Process of the RBQ‐3

The present study followed the steps suggested by Hambleton and Patsula (1998) for scale adaptation in order to adapt the RBQ‐3 into Turkish. The necessary permissions were obtained from the scale developers first for the adaptation. Then, a very detailed translation process was carried out.

After the permission was obtained, the scale items were translated into Turkish. The translation was carried out by researchers with a strong command of English and Turkish in the fields of special education, psychological counseling and guidance, and measurement and evaluation. A rubric was prepared to check the appropriateness of the translations. A section was added to this rubric that allows suggestions for translations deemed inappropriate. The rubric was shared with a researcher with a PhD, an assistant professor, and a professor, all specializing in autism. The expert opinions were examined, and the scale items and instructions were translated into Turkish. The translated RBQ‐3 was then back‐translated and checked by the experts who developed the scale. To ensure linguistic equivalence, coordinated work was conducted with the scale developers.

#### 2.1.3 Procedures

Responses to the self‐report and informant‐report forms of the RBQ‐3 were collected from different study groups in the current research via an online Google Forms. During the data collection phase, support was received from academics (for neurotypical individuals) and ASD associations in Türkiye (for ASD individuals). The data collection periods varied across studies (e.g., 2 months for Study 2) and lasted more than 1 year overall.

Ethical considerations were addressed across all study groups in the present research. Informed consent was obtained from all participants through their agreement on the first page of the online questionnaires. Participants were informed that their participation was voluntary, that they could withdraw at any time, and that the collected data would be used solely for scientific purposes and would not reveal any personal information. Ethical approval for the present study was obtained from the institutional ethics committee of the authors' institution (Approval No: 2024/010).

#### 2.1.4 Statistical Analysis

The factor structure of the RBQ‐3, based on the RBQ‐2, varies across samples, including autistic and neurotypical groups (see Tables 1, 2). Evidence further indicates that RBQ measures (RBQ‐2, RBQ‐2A, and RBQ‐3), which assess RRBs, may have different factor structures depending on the clinical and age composition of the sample (e.g., Jones et al. 2024). Therefore, robust factor‐analytic methods are required to identify the RBQ‐3 factor structure.

Exploratory Structural Equation Modeling (ESEM; for Study 1, 3) is especially useful for identifying factor structure. ESEM is a statistical method that combines Structural Equation Modeling (SEM) and Exploratory Factor Analysis. It allows the use of both exploratory and confirmatory approaches together, especially when performing factor analysis. Therefore, ESEM offers researchers a hybrid approach. This hybrid approach often produces models that represent data more accurately than those based on Confirmatory Factor Analysis (CFA; for Study 1, 3–5) (Gegenfurtner 2022; Swami et al. 2023).

All items in the measurement tool are modeled by associating them with all factors, thereby allowing for a more accurate determination of structural relationships among factors in ESEM models (Asparouhov and Muthén 2009; Prokofieva et al. 2023). ESEM provides better modeling of structure at multidimensional scales (Marsh et al. 2013). Therefore, it can be used effectively for scale development, scale adaptation, discovery of factor structure, and validation of the discovered structure (Marsh et al. 2013; Marsh et al. 2014). Correlations between factors were allowed; all observed variables were permitted to load freely on each factor, and geomin (oblique) rotation was used in our study. Also, because the RBQ‐3 items are ordinal, the ESEM analyses were conducted using the weighted least squares mean and variance adjusted (WLSMV) estimator, as implemented in previous research (Brett et al. 2024; Muthén and Muthén 2009).

Goodness‐of‐fit indices are evaluated for both CFA and ESEM models. The chi‐square ($\chi^{2}$), the comparative fit index (*CFI*), the root mean square error of approximation (*RMSEA*) and the standardized root mean square residual (*SRMR*) are the most commonly reported and interpreted outputs from Mplus. In evaluating these statistics, the following are taken into account (DiStefano and Hess 2005; Schermelleh‐Engel et al. 2003): $\chi^{2}$ value must be insignificant; *CFI* and *TLI* must be 0.95 or higher, *RMSEA* can be maximum of 0.08, and *SRMR* can be maximum of 0.10. Additionally, a non‐significant $\chi^{2}$ generally indicates good model fit; however, $\chi^{2}$ increases with sample size and may be significant even for minor model‐data discrepancies in moderate to large samples (Kline 2011).

Bayesian Confirmatory Factor Analysis (BCFA; for Study 2), which provides statistical evidence for construct validity by testing the hypothesized factor structure in small samples, was performed (Muthén and Asparouhov 2012). The Bayesian approach produces accurate predictions in small samples when appropriate prior distributions are applied and facilitates the evaluation of model‐data fit (Lüdtke et al. 2021). After BCFA, *the posterior predictive p* (*ppp*) value is obtained. This value, around 0.50, is an important measure of a model's accuracy (Gelman et al. 1996; Hoofs et al. 2018; Muthén and Asparouhov 2012). The *ppp* value is a strong indicator of the model's accuracy in small samples (Hoofs et al. 2018). With this value, the 95% *Confidence Interval for the Difference Between the Observed and the Replicated Chi‐Square Values* is also evaluated. The fact that this interval contains zero is considered an indicator of the model's good fit (Alamri 2019).

**FIGURE 1:**
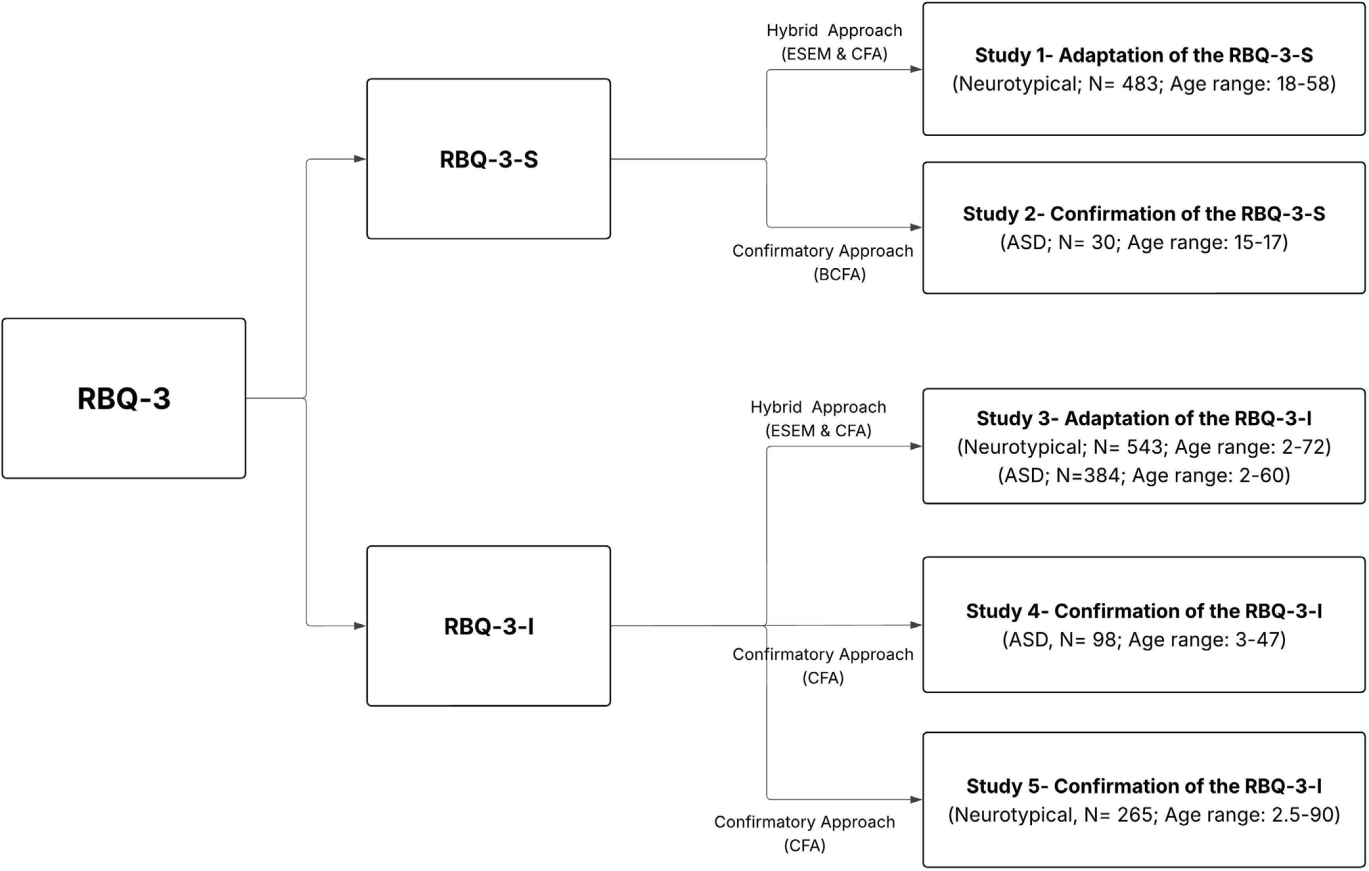
*An Overview of Factor‐Analytic Methods and Sample Characteristics Across Studies*. ESEM: Exploratory Structural Equation Modeling, CFA: Confirmatory Factor Analysis, BCFA: Bayesian Confirmatory Factor Analysis, N: Number of participants. Figure 1 shows that a hybrid approach combining ESEM and CFA was used in Studies 1 and 3, whereas a purely confirmatory approach (BCFA, CFA) was used in Studies 2, 4, and 5. All study groups were independent of one another. The age ranges indicate the years.

In summary, the present study examined the psychometric properties of the Turkish forms of the RBQ‐3 using multiple factor‐analytic methods. These analyses were conducted across five distinct samples with differing characteristics. Figure 1 summarizes the factor analysis methods and sample characteristics for each study included in our research.

Convergent and discriminant validity studies (for Study 1, 3) have been conducted as additional evidence for the construct validity of the RBQ‐3‐S and RBQ‐3‐I. Convergent validity demonstrates that the items are related to the factors they belong to (Fornell and Larcker 1981). Discriminant validity shows that a construct or variable is different from other constructs or variables (Hair et al. 2019). An item is expected to have a low correlation with factors it does not belong to. The average variance extracted (AVE) values for the convergent validity evidence of the scores obtained from the RBQ‐3 and the square roots of the AVE values for discriminant validity evidence were calculated. An AVE value of at least 0.50, ideally at least 0.70, indicates that convergent validity is achieved (Hair et al. 2019, 663). If the square roots of the AVE values are higher than the correlation values calculated between the factors, this indicates that discriminant validity is achieved (Fornell and Larcker 1981; Hair et al. 2019).

Measurement invariance was considered additional important evidence for the construct validity of the RBQ‐3‐I (for Study 3). Measurement invariance assesses whether an instrument measures the same construct consistently across different groups, such as gender, cultural backgrounds, or ethnicities (Rutkowski and Svetina 2014; Schmitt and Kuljanin 2008; Vandenberg and Lance 2000). If measurement invariance is not established, differences in measurements obtained from the same instrument across two groups may be attributed not to true differences, but to the scale measuring different constructs in those groups. This raises a validity problem. Measurement invariance studies should be considered an important part of validity research. Multiple‐Group CFA is conducted to determine measurement invariance, and evidence of invariance is investigated through configural, metric, and scalar invariance stages (Byrne and Stewart 2006).

After ensuring measurement invariance, Welch's *t*‐test (for Study 3) was conducted to examine whether informant‐reported the RBQ‐3‐I scores differed significantly between individuals with ASD and neurotypical individuals. This test is used to investigate differences in scale scores between two independent groups (e.g., an ASD and a neurotypical group) and does not require equal variances between the groups (Delacre et al. 2017). Welch's *t*‐test was conducted to examine whether informant‐reported the RBQ‐3‐I scores differed significantly between the ASD and neurotypical groups, considering both the total scale scores and Item 20, which was excluded from the total score.

Furthermore, the RBQ‐3‐I was completed for individuals from different age groups. The differences between age groups were considered an important source of variance that needed to be accounted for. Therefore, Multiple‐Group Structural Equation Modeling (MG‐SEM; for Study 3) was also conducted with age as a covariate variable in the structural model. We used WLSMV estimation for our ordinal data and tested age‐effect differences using Wald tests, following established procedures (Kline 2011; Muthén and Muthén 2017). MG‐SEM is an extended multi‐group analysis method that allows for the comparison of structural parameters (regression coefficients, factor means, variances) across groups, using measurement invariance as a prerequisite (Byrne 2004; Evermann 2010; Wang 2022). Under the assumption that the measurement model is invariant, it is used to test whether the effects of covariates, such as age, on latent factors differ across groups. In other words, tests of measurement invariance should precede tests of structural invariance. Only when the measurement instrument is operating equivalently across groups can researchers meaningfully test hypotheses about group differences in latent means or structural relationships (Evermann 2010; Putnick and Bornstein 2016; Vandenberg and Lance 2000).

For the reliability of the scores obtained from the RBQ‐3, Cronbach's alpha, McDonald's omega, and Composite Reliability (CR) coefficients were also calculated for all five studies. In general, Cronbach's alpha and McDonald's omega coefficients of at least 0.70 indicate that the scale scores are consistent (Hair et al. 2019). A CR value above 0.60 indicates internal consistency (Fornell and Larcker 1981).

Mplus 8.0 was used to estimate a range of structural models, including ESEM and CFA models, as well as the BCFA model in this research. In addition, measurement invariance and MG‐SEM analyses were conducted using the same software. Jamovi 2.3.28, on the other hand, was employed to assess the reliability of the obtained scores and to perform Welch's *t*‐test analyses.

### 2.2 Study‐Specific Methods

This section describes the methods employed for each of the five study groups included in the research. The specific objective of each group is also outlined. Additionally, information regarding participant recruitment and general characteristics, along with the statistical approaches used to analyze the data, is provided for each group.

#### 2.2.1 Study 1

##### 2.2.1.1 Participants and Procedure

The main objective of Study 1 is adaptation of the RBQ‐3‐S into Turkish using a sample of neurotypical individuals. For this purpose, neurotypical individuals were reached in accordance with the purpose of the RBQ‐3‐S in Study 1. These individuals, most of whom were university students, were expected to complete the RBQ‐3‐S themselves. For the recruitment process, the questionnaire was distributed online via the authors' professional networks, with participants provided with instructions for completing it.

##### 2.2.1.2 Statistical Analysis

The factor structure of the RBQ‐3‐S was investigated using a hybrid approach, with different factor structures also being considered in neurotypical adults. For this purpose, the factor structures shown in (Table 1) were tested with CFA. In addition, due to differences in the RBQ‐3‐S factor structure, the RBQ‐3‐S structure in the Turkish sample was investigated using ESEM, a hybrid exploratory‐confirmatory approach as seen (Figure 1). Two‐factor and four‐factor ESEM were conducted in accordance with the RBQ literature. Additionally, convergent and discriminant validity studies were conducted as further evidence of the construct of the RBQ‐3‐S.

#### 2.2.2 Study 2

##### 2.2.2.1 Participants and Procedure

Study 2 sought to confirm, within a sample of individuals with ASD, the factor structure of the Turkish form of the RBQ‐3‐S that had previously been established in a Turkish neurotypical sample in Study 1. In line with this objective, high school students were reached. These students were diagnosed with ASD according to DSM‐IV criteria by child and adolescent psychiatrists at state hospitals. All participants attended special vocational high schools and were officially placed by Guidance and Research Centers affiliated with the Turkish Ministry of National Education (MoNE) to acquire vocational skills Special Education Services Regulation (Özel Eğitim Hizmetleri Yönetmeliği 2018). For the recruitment process, the authors first obtained permission from the MoNE and then sent the questionnaire online to administrators of special vocational education schools. The school principals granted permission for the study to be conducted and assisted in distributing the consent forms to the parents and collecting them. Then, school administrators were instructed to have students who could read and comprehend the scale and were on the autism spectrum complete it in the school environment. Administrators ensured that the scale was completed by eligible students enrolled in their schools within a two‐week period.

##### 2.2.2.2 Statistical Analysis

Due to the small number of participants in Study 2, the four‐factor ESEM (excluding item 7) factor structure of the RBQ‐3‐S was considered in Study 1. With a confirmatory approach, BCFA was applied to determine the construct validity of the RBQ‐3‐S for individuals with ASD. For this purpose, the factor loadings from Study 1 were used as informative priors in the analysis.

#### 2.2.3 Study 3

##### 2.2.3.1 Participants and Procedure

The purpose of Study 3 is to adapt the RBQ‐3‐I into Turkish based on reports provided by neurotypical informants. To this end, a large sample of informants was recruited to complete the informant form of the RBQ‐3 for both individuals with ASD and neurotypical individuals. For individuals with ASD, parents, caregivers, and teachers who know these individuals well and/or have closely observed their behavior over the past two weeks served as informants, as indicated in the RBQ‐3 manual (Cardiff University 2025). For the recruitment process of individuals with ASD, the authors reached parents/caregivers by distributing the scale online through ASD associations established by families and reached teachers by sending the scale online to special education schools, special education and rehabilitation centers, and guidance and research centers. For neurotypical individuals, the RBQ‐3‐I was primarily completed by university students for individuals they knew well, as per the RBQ‐3 manual. The authors distributed the scale online via professional networks and provided instructions for completing it.

##### 2.2.3.2 Statistical Analysis

Study 3 employed a hybrid approach to examine the factor structure of the RBQ‐3‐I as seen in Figure 1. The factor structures of the RBQ‐3‐I shown in Table 2 were tested using CFA. Furthermore, the factor structure of the RBQ‐3‐I in the Turkish sample was also investigated using two‐factor and four‐factor ESEM in accordance with the RBQ literature. Additional studies regarding the construct validity of the RBQ‐3‐I have been performed. First, convergent and discriminant validity studies were conducted. Second, measurement invariance was tested to provide evidence of construct validity across samples with different characteristics. A Welch's *t*‐test was carried out after establishing measurement invariance. Additionally, MG‐SEM was examined using scores obtained from the RBQ‐3‐I administered to different age groups in Study 3.

#### 2.2.4 Study 4

##### 2.2.4.1 Participants and Procedure

The aim of Study 4 is to confirm the Turkish version of the RBQ‐3‐I using informant reports (e.g., from teachers and parents) on a sample of individuals with ASD. The factor structure of this scale was previously established in Study 3. Participants of Study 4 completed the RBQ‐3‐I for individuals they knew well who had been officially diagnosed with ASD by child and adolescent psychiatrists at state hospitals. Parents/caregivers and other relatives were recruited through ASD family associations via an online scale, whereas teachers were recruited from special education schools, special education and rehabilitation centers, and guidance and research centers.

##### 2.2.4.2 Statistical Analysis

Study 4 investigated the factor structure of the RBQ‐3‐I using CFA. The model tested was the four‐factor ESEM model, with items 1 and 9 excluded. This model had previously been identified as the most appropriate structure for the Turkish sample in Study 3.

#### 2.2.5 Study 5

##### 2.2.5.1 Participants and Procedure

Study 5 aimed to confirm, using informant reports (e.g., about their mothers, fathers, or siblings) on a sample of neurotypical individuals, the factor structure of the Turkish version of the RBQ‐3‐I that was previously established in Study 3. A new study group was recruited to investigate the construct validity of the RBQ‐3‐I in neurotypical university students. For the recruitment process, the authors shared the scale online with their professional networks, along with instructions on how and for whom it should be completed.

##### 2.2.5.2 Statistical Analysis

Based on Study 3's findings, the four‐factor ESEM structure of the RBQ‐3‐I (excluding item 1 and item 9) was selected as the most adequate model for the Turkish sample. This structure was subsequently re‐examined in Study 5 using a CFA.

## 3 Results

### 3.1 Study 1

#### 3.1.1 Participants

Study 1 included 483 participants, of whom 18.20% were male and 81.80% were female. The mean age of participants was 28.85 years (SD = 7.96, age range: 18–58). The majority of the participants were university students (82.00%), while 8.70% had postgraduate education. Smaller proportions of participants completed high school (6.80%), middle school (0.80%), or primary school (1.70%).

#### 3.1.2 Statistical Findings

##### 3.1.2.1 Validity and Reliability of the Turkish Form of the RBQ‐3‐S

The results of CFA and ESEM conducted for the Turkish form of the RBQ‐3‐S are presented in Table 3. The factor loadings obtained from the models Table S1 to Table S8. Also, correlations between factors are available in the Tables S1.1, S2.1, … and S8.1.

**TABLE 3:** Model fit indices for the RBQ‐3‐S Turkish form from the CFA and ESEM for study 1.

Factor analytic method	Chi‐square (df)	RMSEA [90% CI]	SRMR	CFI	TLI
2‐Factor CFA Barrett et al. (2015)	312.67* (76)	0.08* [0.07–0.09]	0.07	0.94	0.93
2‐Factor CFA (Barrett et al. 2018)	576.44* (76)	0.09* [0.08–0.09]	0.08	0.90	0.88
4‐Factor CFA (Brett et al. 2024)**	272.16* (113)	0.05* [0.05–0.06]	0.05	0.97	0.96
2‐Factor ESEM	416.94* (134)	0.07* [0.06–0.07]	0.05	0.95	0.94
2‐Factor ESEM (Excluding Item 9)	330.32* (118)	0.06* [0.05–0.07]	0.05	0.96	0.95
2‐Factor ESEM (Excluding Item 9 and Item 17)	302.96* (103)	0.06* [0.06–0.07]	0.05	0.96	0.94
4‐Factor ESEM	230.11* (101)	0.05* [0.04–0.06]	0.04	0.98	0.96
4‐Factor ESEM (Excluding Item 7)	**205**.14* (**87**)	0.05* [**0.04**–**0.06**]	**0.04**	**0.98**	**0.96**

*Note:* CFI: comparative fit index; CI: confidence interval; RMSEA: root mean square error of approximation; TLI: Tucker Lewis index.

*p < 0.05.

**Attention was paid to its general factor structure, but item number 20 was not included; the model indicated in bold is the model that shows the best fit.

The models considered for the Turkish form of the RBQ‐3‐S generally demonstrated good fit. However, the best fit was achieved with the four‐factor ESEM (excluding item 7). In this model, item 7 was removed because it had cross‐loadings on multiple factors (for a detailed examination of factor loadings, see Table S7). This model is largely similar to the model of Brett et al. (2024). Table 4 presents the reliability estimates for the four‐factor ESEM (excluding item 7) structure. Reliability estimates for the other models can be found in Table S9.

**TABLE 4:** Reliability of scores obtained from the four‐factor ESEM (excluding item 7) of the RBQ‐3‐S for study 1.

Factors	Cronbach's Alpha	McDonald's Omega
F1: Repetitive motor behaviors (items: 1, 2, 3, 4, 5, 6)	0.78	0.79
F2: Interest in sensations & objects (items: 8, 9, 10, 11, 12)	0.76	0.77
F3: Insistence on sameness (items: 13, 14, 15, 16)	0.79	0.80
F4: Restricted interests (items: 17, 18, 19)	0.67	0.69
Total	0.89	0.88

Based on the four‐factor ESEM model (excluding item 7), the reliability estimates for the factor scores and the total scale score of the RBQ‐3‐S were 0.70 or above for all factors and the total score, except for F4. The scale scores are expected to have a reliability of at least 0.70. However, the number of items affects reliability estimates; as the number of items increases, reliability values also increase (Mehrens and Lehmann 1991). In addition, reliability estimates obtained in Study 1 are consistent with those reported by Brett et al. (2024).

##### 3.1.2.2 Convergent and Discriminant Validity of the Turkish Form of the RBQ‐3‐S

For convergent and discriminant validity, a CFA was performed using the four‐factor‐ESEM (excluding item 7) factor structure, which is valid for the Turkish form of the RBQ‐3‐S. The results obtained for CFA were $\chi^{2}$_(129)_ = 306.80, *p* = 0.00; **CFI** = **0**.**97**; **TLI** = **0**.**96**; **RMSEA** = **0**.**05** (95% CI [0.05–0.06]); **SRMR** = **0**.**05** (bold values indicate that the model is acceptable). AVE results obtained from the factor loadings after CFA were calculated as 0.57 for F1, 0.50 for F2, 0.58 for F3, and 0.55 for F4. Since AVE values were 0.50 or higher, convergent validity was ensured. The correlation values between the factors were r_F1‐F2_ = 0.77, r_F1‐F3_ = 0.53, r_F1‐F4_ = 0.67, r_F2‐F3_ = 0.66, r_F2‐F4_ = 0.68, and r_F3‐F4_ = 0.75. Since the square roots of the AVE values range from 0.71 to 0.76, it can be concluded that discriminant validity is achieved. In addition, CR values were calculated as 0.88 for F1, 0.83 for F2, 0.85 for F3, and 0.79 for F4. Accordingly, internal consistency is high.

### 3.2 Study 2

#### 3.2.1 Participants

Study 2 comprised 30 high school students. The gender distribution of the sample was 66.67% male and 33.33% female. The mean age of the participants was 16.20 years (SD = 0.76), with ages ranging between 15 and 17 years.

#### 3.2.2 Statistical Findings

##### 3.2.2.1 Validity and Reliability of the Turkish Form of the RBQ‐3‐S

As a result of BCFA, the *ppp* value was determined as 0.31, and the 95% CI *Chi‐Square Difference* was [−54.18–88.02]. It is stated that the *ppp* values obtained after BCFA range between 0 and 1, with values close to 0.50 indicating excellent model fit; values below 0.10 or above 0.90 indicating a lack of fit, and values between these ranges (0.10–0.90) being considered acceptable in terms of model fit (Dombrowski et al. 2018). The fact that the 95% CI *Chi‐Square Difference* range included zero and the *ppp* value was between 0.10 and 0.90 indicates that the model fits the data. Table 5 shows the reliability estimates of the RBQ‐3‐S from Study 2.

**TABLE 5:** Reliability of scores obtained from the Four‐Factor ESEM (excluding item 7) of the RBQ‐3‐S for study 2.

Factors	Cronbach's Alpha	McDonald's Omega
F1: Repetitive motor behaviors (items: 1, 2, 3, 4, 5, 6)	0.69	0.75
F2: Interest in sensations & objects (items: 8, 9, 10, 11, 12)	0.74	0.75
F3: Insistence on sameness (items: 13, 14, 15, 16)	0.86	0.87
F4: Restricted interests (items: 17, 18, 19)	0.76	0.78
Total	0.88	0.87

As shown in Table 5, the RBQ‐3‐S scores demonstrated satisfactory reliability. Compared with the estimates in Table 4, the reliability values were similar. Overall, these findings indicate that the RBQ‐3‐S yields reliable scores across the different samples examined.

### 3.3 Study 3

#### 3.3.1 Participants

A total of 927 participants contributed to Study 3 by completing the informant version of the RBQ‐3. These informants reported on two distinct groups: individuals with ASD and neurotypical individuals. Table 6 provides a summary of the demographic characteristics of both groups as derived from the informant reports.

**TABLE 6:** Demographic Characteristics of sample 3.

Variables	Individuals with ASD	Neurotypical individuals
**Age**	**Mean (SD)**	**Mean (SD)**
	11.49 (9.16)	25.27 (11.39)
**Gender**	***f* (%)**	***f* (%)**
Female	152 (28.00)	243 (63.30)
Male	390 (71.80)	141 (36.70)
Non‐specified	1 (0.20)	—
**Education level**	***f* (%)**	***f* (%)**
Non‐specified	1 (0.20)	—
Non‐literate	249 (45.90)	27 (7.00)
Primary	153 (28.20)	23 (6.00)
Secondary	82 (15.10)	25 (6.50)
High school	58 (10.70)	46 (12.00)
Bachelor's degree	—	241 (62.80)
Master's degree	—	22 (5.70)
Total	543 (100.00)	384 (100.00)

Table 6 summarizes that most individuals with ASD are male (71.80%) and non‐literate (45.90%). In contrast, most neurotypical individuals are female (63.30%) and have at least a bachelor's degree (62.80%). The average ages of individuals with ASD (Mean = 11.49, SD = 9.16, age range: 2–60) and neurotypical individuals (Mean = 25.27, SD = 11.39, age range: 2–72) in Sample 3 are noticeably different.

When examining participants' responses in Study 3 regarding whom they completed the scale for, most did not answer this item. However, among those who completed the scale for individuals with ASD, 20.1% completed it for their student, 13.3% for a relative (cousin), 5.7% for their son, 1.5% for their sibling, and 0.9% for their daughter. Among those who completed the scale for neurotypical individuals, 36.8% completed it for a relative (cousin), 3.9% for their student, 1.3% for their sibling, and 0.8% for their son.

#### 3.3.2 Statistical Findings

##### 3.3.2.1 Validity and Reliability of the Turkish Form of the RBQ‐3‐I

The results of CFA and ESEM conducted for the Turkish form of the RBQ‐3‐I are presented in Table 7. The factor loadings obtained from the Table S10 to Table S17. Also, correlations between factors are in Tables S10.1, S11.1, … and S17.1.

**TABLE 7:** Model fit indices for the RBQ3‐I Turkish form from the CFA and ESEM for study 3.

Factor analytic method	Chi‐square (df)	RMSEA [90% CI]	SRMR	CFI	TLI
2‐Factor CFA (Lidstone et al. 2014; Uljarević, Arnott, et al. (2017); Uljarević, Richdale, et al. 2017)	1615.17* (118)	0.12* [0.11–0.12]	0.07	0.91	0.90
2‐Factor CFA (Leekam et al. 2007)	1123.75* (118)	0.09* [0.09–0.10]	0.06	0.94	0.93
2‐Factor ESEM	988.83* (134)	0.08* [0.08–0.09]	0.04	0.96	0.94
2‐Factor ESEM (Excluding Item 10)	835.09* (118)	0.08* [0.07–0.08]	0.04	0.96	0.95
2‐Factor ESEM (Excluding Item 10 and Item 1)	737.56* (103)	0.08* [0.07–0.08]	0.04	0.96	0.95
4‐Factor ESEM	473.05* (101)	0.06* [0.06–0.07]	0.02	0.98	0.97
4‐Factor ESEM (Excluding Item 1)	377.72* (87)	0.06* [0.05–0.07]	0.02	0.98	0.97
4‐Factor ESEM (Excluding Items 1 and Item 9)	**267**.30* (**74**)	0.05* [**0**.**05–0**.**06**]	**0.02**	**0.99**	**0.98**

*Note:* CFI: comparative fit index; CI: confidence interval; RMSEA: root mean square error of approximation; TLI: Tucker Lewis index. The model indicated in bold is the model that shows the best fit.

*p < 0.05.

The models considered for the Turkish form of the RBQ‐3‐I generally demonstrated good fit. However, the best fit was achieved with the four‐factor ESEM (excluding item 1 and item 9). While item 1 was generally removed in previous studies, a better model was achieved by removing item 9 in the Turkish sample. Table 8 shows the reliability estimates for the four‐factor ESEM (excluding item 1 and item 9) structure. Reliability estimates for other models can be found in Table S18.

**TABLE 8:** Reliability of scores obtained from the four‐factor ESEM (excluding item 1 and item 9) of the RBQ‐3‐I for study 3.

Factors	Cronbach's Alpha	McDonald's Omega
F1: Repetitive motor behaviors (items: 2, 3, 4, 5, 6)	0.87	0.88
F2: Interest in sensations & objects (items: 7, 8, 10, 11, 12)	0.81	0.81
F3: Insistence on sameness (items: 13, 14, 15, 16)	0.84	0.84
F4: Restricted interests (items: 17, 18, 19)	0.73	0.73
Total	0.92	0.92

Based on the four‐factor ESEM model of the RBQ‐3‐I (excluding item 1 and item 9), reliability estimates for both the factor scores and the total scale score were 0.70 or higher. These findings indicate adequate internal consistency of the scores obtained from the RBQ‐3‐I. Accordingly, the scores derived from the scale are reliable.

##### 3.3.2.2 Convergent and Discriminant Validity of the Turkish Form of the RBQ‐3‐I

Convergent and discriminant validity evidence for the Turkish form of the RBQ‐3‐I was investigated based on CFA results performed for the four‐factor ESEM (excluding item 1 and item 9) model. The results obtained for CFA were $\chi^{2}$_(113)_ = 659.84, *p* = 0.00; **CFI** = **0**.**97**; **TLI** = **0**.**96**; **RMSEA** = **0**.**07** (95% CI [0.07–0.08]); **SRMR** = **0**.**04** (bold values indicate that the model is acceptable). AVE results obtained from the factor loadings after CFA were calculated as 0.69 for F1, 0.57 for F2, 0.66 for F3, and 0.56 for F4, indicating that convergent validity was ensured since all AVE values were 0.50 and above. The correlation values between factors were r_F1‐F2_ = 0.82, r_F1‐F3_ = 0.59, r_F1‐F4_ = 0.74, r_F2‐F3_ = 0.75, r_F2‐F4_ = 0.84, and r_F3‐F4_ = 0.82. Given that the square roots of the AVE values range from 0.75 to 0.83, discriminant validity can be considered marginally achieved. In addition, CR values were calculated as 0.92 for F1, 0.87 for F2, 0.88 for F3, and 0.79 for F4. Thus, internal consistency is high.

##### 3.3.2.3 Measurement Invariance of the Turkish Form of the RBQ‐3‐I

The RBQ‐3‐I was administered to a large sample in the present study. The model that yielded the best results for this study group was the four‐factor structure, with items 1 and 9 removed. Measurement invariance was investigated on this model using data from informants who provided responses for individuals with ASD and for neurotypical individuals. The results obtained are shown in Table 9.

**TABLE 9:** Results of measurement invariance of the RBQ‐3‐I Turkish form for study 3.

	Chi‐square (df)	RMSEA [90% CI]	CFI	TLI	SRMR	ΔSRMR	ΔRMSEA	ΔCFI	ΔTLI
ASD individuals	440.57* (113)	0.073* [0.07–0.08]	0.95	0.95	0.04	—	—	—	—
Neurotypical individuals	266.68* (113)	0.060* [0.05–0.07]	0.98	0.98	0.05	—	—	—	—
Configural	709.58* (226)	0.068* [0.06–0.07]	0.97	0.96	0.05	—	—	—	—
Metric	796.11* (239)	0.071* [0.06–0.07]	0.96	0.96	0.05	0.002	0.003	−0.005	−0.003
Scalar	825.34* (269)	0.067* [0.06–0.07]	0.96	0.96	0.05	0.001	−0.004	0.000	0.005

*Note:* Δ: change compared to previous model; CFI: comparative fit index; RMSEA: root mean square error of approximation; TLI: Tucker Lewis index.

*p < 0.05.

The model must be fitted separately for the informant‐reported data on the ASD group and the informant‐reported data on the neurotypical group for measurement invariance. The four‐factor structure, with items 1 and 9 removed, demonstrated good fit for both groups. Then, configural, metric, and scalar invariance were tested as in Brett et al. (2024). According to the test results, ΔSRMR, ΔRMSEA, ΔCFI, and ΔTLI values range from −0.01 to 0.01. This shows that measurement invariance is achieved (Cheung and Rensvold 2002). Accordingly, the RBQ‐3‐I measures individuals with different characteristics (ASD and neurotypical) in a similar structure.

It was concluded that the RBQ‐3‐I demonstrates measurement invariance for informant‐reported data across both ASD and neurotypical individuals, indicating that the scale does not favor one informant group over the other and accurately measures the intended construct. In addition, the scores obtained from these groups were compared using Welch's *t*‐test. Respectively; for F1 (Welch's *t*_(901)_ = −14.41, *p* < 0.001, Cohen's *d* = −0.95); for F2 (Welch's *t*_(885)_ = −11.41, *p* < 0.001, Cohen's *d* = −0.75); for F3 (Welch's *t*_(887)_ = −8.30, *p* < 0.001, Cohen's *d* = −0.55); for F4 (Welch's *t*_(875)_ = −16.52, *p* < 0.001, Cohen's *d* = −1.09), and for the entire scale (Welch's *t*_(874)_ = −15.13, *p* < 0.001, Cohen's *d* = −1.00). The scores obtained from the informant‐reported data show statistically significant differences between ASD and neurotypical individuals and are higher for individuals with ASD.

A comparison was also made for item 20 of the RBQ‐3‐I. This item is a three‐point Likert‐type item and is not included in the scale's total score. However, it is important in terms of providing information about repetitive behavior to scale implementers. In the comparison for this item, a higher item score was observed for ASD individuals (Welch's *t*_(808)_ = −13.00, *p* < 0.001, Cohen's *d* = −0.87).

The construct validity of the RBQ‐3‐I Turkish form has been established for both ASD and neurotypical individuals. However, as shown in Table 6, the mean ages of individuals with ASD (Mean = 11.49, SD = 9.16) and neurotypical individuals (Mean = 25.27, SD = 11.39) are quite different. Therefore, the study also conducted MG‐SEM with age as a covariate variable in the structural model. The MG‐SEM model with age as a covariate showed a good fit. The results obtained for CFA were $\chi_{\left(295\right)}^{2}$= 790.47, *p* = 0.00; **CFI** = **0**.**97**; **TLI** = **0**.**97**; **RMSEA** = **0**.**06** (95% CI [0.06–0.07]); **SRMR** = **0**.**05** (bold values indicate that the model is acceptable). The MG‐SEM model results revealed that the effect of age on the factors did not differ significantly between the ASD and neurotypical groups (Wald *χ* ^2^ = 7.29, df = 4, *p* = 0.12). This result suggests that the scale measures the factors consistently across ASD and neurotypical groups, regardless of age differences between groups.

### 3.4 Study 4

#### 3.4.1 Participants

A total of 98 participants took part in Study 4. Among the participants, 51.0% completed the form for their sons, 26.5% for their students, 19.4% for their daughters, and 3.1% for other relatives (e.g., cousins). Of individuals with ASD, 72.40% were male and 27.60% were female. The mean age of individuals with ASD was 13.58 years (SD = 10.18, *age range*: 3–47), and most individuals with ASD are non‐literate (43.90%). Also, individuals with ASD were 27.60% at primary school, 17.30% at high school, 9.20% at middle school, and only 2.00% at university.

#### 3.4.2 Statistical Findings

##### 3.4.2.1 Validity and Reliability of the Turkish Form of the RBQ‐3‐I

The obtained results are $\chi^{2}$_(113)_ = 163.59, *p* = 0.00; **CFI** = **0**.**96**; **TLI** = **0**.**95**; **RMSEA** = **0**.**07** (95% CI [0.04–0.09]); **SRMR** = **0**.**07** (bold values indicate that the model is acceptable). Accordingly, the four‐factor ESEM model for the Turkish form of the RBQ‐3‐I (excluding items 1 and 9) was confirmed in a separate study group. The reliability of the scores obtained from this group was determined as follows, respectively, for F1 *α* = 0.84, *ω* = 0.84; For F2 *α* = 0.80, *ω* = 0.81; for F3 *α* = 0.75, *ω* = 0.76; for F4 *α* = 0.55, *ω* = 0.59, and for the entire scale *α* = 0.89, *ω* = 0.90. The scores obtained reliable results except for F4. It is thought that the most important reason for reaching low reliability values in this dimension is the number of items.

### 3.5 Study 5

#### 3.5.1 Participants

Study 5 comprised 265 participants who completed the informant form of the RBQ‐3. Of the participants, 35.1% completed the scale for their mothers, 16.2% for their siblings, and 14.0% for their fathers. Of the individuals about whom information was provided, 60.4% were female and 39.6% were male. The mean age of the individuals for whom the scale was completed was 38.20 years (SD = 17.88; *age range*: 2.5–90 years). Their educational levels were as follows: university education (27.2%), primary and secondary school (25.7%), middle school (10.9%), nonliterate (6.4%), and postgraduate education (4.2%).

#### 3.5.2 Statistical Findings

##### 3.5.2.1 Validity and Reliability of the Turkish Form of the RBQ‐3‐I

The obtained results are $\chi^{2}$_(113)_ = 160.16, *p* = 0.00; **CFI** = **0**.**98**; **TLI** = **0**.**98**; **RMSEA** = **0**.**04** (95% CI [0.02–0.05]); **SRMR** = **0**.**05** (bold values indicate that the model is acceptable). These values, obtained after CFA, confirm the factor structure of the RBQ‐3‐I form when applied to a new neurotypical study group. The reliability values obtained from Study 5 were as follows: for F1 *α* = 0.79, *ω* = 0.81; for F2 *α* = 0.83, *ω* = 0.84; for F3 *α* = 0.84, *ω* = 0.84; for F4 *α* = 0.68, *ω* = 0.68, and for the entire scale *α* = 0.903, *ω* = 0.91. Similar to Study 4, the reliability estimates were high for all dimensions except F4.

## 4 Discussion

The primary objective of the present study was to adapt the RBQ‐3, a scale derived from the RBQ‐2 and the RBQ‐2A, both of which have been established as valid and reliable measures of RRBs, into the Turkish context. The RBQ‐2 and the RBQ‐2A are measurement tools that are considered in many studies (Collis et al. 2022; Jacob and Alexander 2023; Joyce et al. 2017; Longo et al. 2022; Moore et al. 2022; Nwaordu and Charlton 2024; Recio et al. 2024; Schulz and Stevenson 2020; Uljarević, Richdale, et al. 2017) due to their shortness and wide application age range (applicable to infants and adults). The RBQ‐3, which preserves the items of the RBQ‐2 and the RBQ‐2A but offers better scaling, was developed by Jones et al. (2024) to assess repetitive behaviors. In fact, unlike the RBQ‐2 and RBQ‐2A, 19 items are structured in an ordered four‐point Likert‐type scale. This condition provides an advantage in determining psychometric properties.

In studies examining the factor structures of the RBQ‐2 and the RBQ‐2A, samples with diverse characteristics have been included. These samples comprised neurotypical adults and university students (Barrett et al. 2015; Brett et al. 2024), as well as adults with ASD (Barrett et al. 2018). In addition, research has involved younger populations, including individuals with ASD aged 2–17 years (Lidstone et al. 2014), typically developing 6‐year‐old children (Uljarević, Arnott, et al. 2017), and typically developing 2‐year‐old children (Leekam et al. 2007).

The current research comprises five different studies with five different samples. Study 1 included university students and adults as participants for the RBQ‐3‐S, whereas Study 2 consisted entirely of high school students with ASD. Study 3 collected the RBQ‐3‐I data from teachers, parents, or close relatives of individuals with ASD. Furthermore, regarding the neurotypical individuals in Study 3, the scale was completed mostly by university students, as well as by adults for people they knew well (e.g., cousins). Study 4 involved parents and teachers of children with ASD who completed the RBQ‐3‐I, whereas Study 5 consisted of university students who completed the scale, most often for their parents. For all studies, the RBQ‐3 data were collected in accordance with the RBQ manual. The manual specifies that responses to the RBQ‐3‐I should be provided with reference to well‐known individuals.

The study involved diverse samples of children, adults, and family members across a wide age range (15–58 years for the RBQ‐3‐S and 2–90 years for the RBQ‐3‐I, with the latter referring to the individuals for whom the scale was completed). This can be considered a meaningful step toward demonstrating that the current adaptation can be used to assess RRBs across diagnostic groups and the lifespan. Additionally, this research takes a step further by obtaining first‐hand information from individuals with ASD during the scale adaptation process, as recommended by Jones et al. (2024).

The Turkish samples of the RBQ‐3‐S and RBQ‐3‐I, unlike those in other studies, are larger and more representative. The Turkish form of the RBQ‐3‐S was adapted with 4 factors: Repetitive Motor Behaviors (Items: 1, 2, 3, 4, 5, 6); Interest in Sensations & Objects (Items: 8, 9, 10, 11, 12); Insistence on Sameness (Items: 13, 14, 15, 16) and Restricted Interests (Items: 17, 18, 19). Item 7 was removed from the Turkish form of the RBQ‐3‐S. This four‐factor structure of the scale is similar to that proposed by Brett et al. (2024). The Turkish form of the RBQ‐3‐I was adapted with 4 factors: Repetitive Motor Behaviors (Items: 2, 3, 4, 5, 6), Interest in Sensations & Objects (Items: 7, 8, 10, 11, 12), Insistence on Sameness (Items: 13, 14, 15, 16) and Restricted Interests (Items: 17, 18, 19). Although this factor structure is very similar to that of the Turkish form of the RBQ‐3‐S. Items 1 and 9 were removed from the Turkish form of the RBQ‐3‐I. Since the items in the factors were quite similar, the factor names were also identical.

Due to the limited number of individuals with ASD reached in the present study, the model fit of the RBQ‐3‐S was examined in the ASD sample using BCFA rather than conventional CFA. To conduct BCFA, the factor loadings from the neurotypical sample were used, and an acceptable model‐data fit was obtained. Given the small ASD sample size, measurement invariance for the RBQ‐3‐S has not yet been investigated, although such an analysis would provide strong evidence for construct validity. Although the current data for the RBQ‐3‐S were obtained from university students, consistent with the literature (Barrett et al. 2015; Brett et al. 2024), future research should examine the factor structure of the RBQ‐3‐S in larger, more heterogeneous adult samples, including individuals with ASD.

In the present study, convergent and discriminant validity evidence for RBQ‐3‐S and RBQ‐3‐I, and measurement invariance for RBQ‐3‐I were investigated with various factor‐analytic methods. These methods are basic techniques in investigating construct validity. Although the RBS‐R and Gilliam Autistic Trait Rating Scale (GARS) scales were not used for criterion‐based validity in the current study due to the large number of items for the Turkish sample, they can be examined in subsequent studies to provide criterion‐based validity evidence.

Given the importance of measuring RRBs in ASD research, easy‐to‐apply and easy‐to‐evaluate measurement tools are essential, even if they are not intended for clinical diagnostic purposes. Of course, the psychometric properties of these tools are indisputably necessary for them to yield valid and reliable results. At this point, the use of short, psychometrically satisfactory tools that teachers, counselors, parents, or individuals without intellectual disabilities can use in their self‐assessment is valuable.

Despite requiring a multidisciplinary approach, the official diagnosis of ASD in Türkiye is made by child and adolescent psychiatrists, and it is suggested that multi‐informant resources should be used for accurate decision‐making (Öner 2020). On the other hand, the emergence of RBQ symptoms may be difficult to assess in clinical settings (Öner 2020). Considering its clinical use, the RBQ‐3 may be a critical tool that can be applied outside of clinical settings by other informants. Since the RBQ‐3 is an easy‐to‐use tool and does not require specific training, it may be possible to train first‐step healthcare personnel, such as family physicians, to identify children at risk for ASD and to refer them to second‐step personnel, such as child and adolescent psychiatrists, for a more comprehensive multidisciplinary assessment in Türkiye. Such assessments are typically characterized by detailed interviews, behavioral observations, and evaluations of ASD‐related characteristics (Jones et al. 2024). Although the RBS‐R and GARS, consisting of 42 items and developed by Gilliam (2006) and adapted to Turkish by Diken et al. (2012), are used to measure RRBs, they consist of a substantial number of items. However, the RBQ‐3‐S and the RBQ‐3‐I contain very similar items to those in these measurement tools; in other words, they measure the same structure. Moreover, the RBQ‐3 has a small number of items, and its psychometric properties are satisfactory for both forms. Therefore, it is a valid, reliable, and useful measurement tool for measuring RRBs. The scale items (original and Turkish forms) can be accessed from Cardiff University (2025).

## 5 Limitations

Despite the substantial number of participants, a major limitation of the study is the limited number of individuals with ASD as primary respondents. The lack of official data on individuals with ASD in Türkiye limits the ability to access this population, particularly those with the literacy skills necessary to complete the RBQ‐3‐S. This limitation has also posed challenges for researchers in reaching the ASD population. Participants who provided data for individuals with ASD were recruited through ASD associations, which facilitated access to families, special education, and rehabilitation centers offering educational support for individuals with special needs, as well as guidance and research centers conducting educational assessments. Although the RBQ‐3 is designed for both children and adults, adults with ASD could not be reached in Türkiye. Nevertheless, the RBQ‐3‐S was administered to a small number of high school students diagnosed with ASD.

Due to the above‐mentioned limitation, a procedure similar to that used for the RBQ‐3‐I was not adopted to investigate the factor structure of the RBQ‐3‐S in the Turkish sample. In other words, in examining the factor structure of the RBQ‐3‐I for the Turkish sample, analyses were initially conducted using a combined dataset of neurotypical individuals and individuals with ASD. Subsequently, two additional studies were carried out: Study 4 with individuals with ASD and Study 5 with neurotypical individuals. Although Studies 1 and 2 provide evidence for the construct validity of the RBQ‐3‐S in the Turkish sample, they were not conducted on a combined sample, unlike the RBQ‐3‐I. Scales should be monitored after they have been developed or adapted. In this context, future research may address this limitation by conducting further construct validity studies of the RBQ‐3‐S on a combined sample. In addition, while the factor structures for RBQ‐3‐S and RBQ‐3‐I identified in this study are robust, further studies are needed to determine whether these structures are invariant across different samples. Researchers are encouraged to test alternative factor structures in their own samples according to literature and compare their findings with the framework presented here. Longitudinal monitoring of the factor structures across different applications will further enhance our understanding of the psychometric properties of the RBQ‐3.

Another major limitation that should be acknowledged is sampling characteristics. Study 1 is a neurotypical sample, but it is also predominantly female, which does not match the sex distribution within ASD. This imbalance may have influenced the findings, as it assumes that repetitive behaviors in neurotypical females are comparable to those in neurotypical males and individuals with ASD, regardless of sex assigned at birth. Relatedly, in Study 3, the measurement invariance of the informant‐report form of the Turkish RBQ‐3 is compared across a neurotypical and an ASD sample. Nevertheless, the samples differ significantly in age, educational attainment, sex distribution, and informant type. These differences may have contributed to the observed variations in measurement invariance, beyond diagnostic status alone. The presence of these potential confounding factors limits the generalizability of the findings. For future research, it is suggested to recruit more demographically balanced samples and to consider subgroup analyses by sex, age, and informant characteristics. In addition, the use of matched samples or statistical controls may help to isolate better the effects of neurotypical versus ASD status on RRBs. Although age was considered as a covariate in this study and found not to have a significant effect on the measurement model, it would be beneficial to work with balanced age‐group samples.

## 6 Conclusion

The findings of the current study demonstrate that the Turkish form of the RBQ‐3 is a valid and reliable questionnaire measuring RRBs across the lifespan. The RBQ‐3 enables both clinicians and researchers to understand RRBs through self—and informant‐report forms better. In conclusion, the Turkish RBQ‐3 has remarkable potential to meet the need for a practical assessment tool for the Turkish ASD population, particularly for understanding and evaluating RRBs. Beyond its application to individuals with ASD, the RBQ‐3 has significant value for assessing RRBs in other clinical conditions and in the wider population. This broader utility contributes to a more comprehensive understanding of the prevalence and manifestation of RRBs across diverse clinical and non‐clinical groups for practitioners and researchers.

## Conflicts of Interest

The authors declare no conflicts of interest.

## Supporting information


**Table S1:** Factor Loadings from the Barrett et al. (2015) Two‐Factor Model Applied to Study 1.
**Table S1.1:** Correlations Between Factors from the Barrett et al. (2015) Two‐Factor Model in Study 1.
**Table S2:** Factor Loadings from the Barrett et al. (2018) Two‐Factor Model Applied to Study 1.
**Table S2.1:** Correlations Between Factors from the Barrett et al. (2018) Two‐Factor Model in Study 1.
**Table S3:** Factor Loadings from the Brett et al. (2024) Four‐Factor Model Applied to Study 1.
**Table S3.1:** Correlations Between Factors from the Brett et al. (2024) Four‐Factor Model in Study 1.
**Table S4:** Factor Loadings from the Two‐Factor ESEM Applied to Study 1.
**Table S4.1:** Correlations Between Factors from the Two‐Factor ESEM in Study 1.
**Table S5:** Factor Loadings from the Two‐Factor ESEM (Excluding Item 9) Applied to Study 1.
**Table S5.1:** Correlations Between Factors from the Two‐Factor ESEM (Excluding Item 9) in Study 1.
**Table S6:** Factor Loadings from the Two‐Factor ESEM (Excluding Item 9 and Item 17) Applied to Study 1.
**Table S6.1:** Correlations Between Factors from the Two‐Factor ESEM (Excluding Item 9 and Item 17) in Study 1.
**Table S7:** Factor Loadings from the Four‐Factor ESEM Applied to Study 1.
**Table S7.1:** Correlations Between Factors from the Four‐Factor ESEM in Study 1.
**Table S8:** Factor Loadings from the Four‐Factor ESEM (Excluding Item 7) Applied to Study 1.
**Table S8.1:** Correlations Between Factors from the Four‐Factor ESEM (Excluding Item 7) in Study 1.
**Table S9:** Reliability of Scores Obtained from the RBQ‐3‐S Turkish Form for Study 1.
**Table S10:** Factor Loadings from the Lidstone et al. (2014) and Uljarević, Arnott, et al. (2017), Uljarević, Richdale, et al. (2017) Two‐Factor Model Applied to Study 3.
**Table S10.1:** Correlations Between Factors from Lidstone et al. (2014), Uljarević, Arnott, et al. (2017) and Uljarević, Richdale, et al. (2017) Two‐Factor Model in Sample 3.
**Table S11:** Factor Loadings from the Leekam et al. (2007) Two‐Factor Model Applied to Study 3.
**Table S11.1:** Correlations Between Factors from the Leekam et al. (2007) Two‐Factor Model in Study 3.
**Table S12:** Factor Loadings from the Two‐Factor ESEM Applied to Study 3.
**Table S12.1** Correlations Between Factors from the Two‐Factor ESEM in Study 3.
**Table S13:** Factor Loadings from the Two‐Factor ESEM (Excluding Item 10) Applied to Study 3.
**Table S13.1:** Correlations Between Factors from the Two‐Factor ESEM in Study 3.
**Table S14:** Factor Loadings from the Two‐Factor ESEM (Excluding Item 10 and Item 1) Applied to Study 3.
**Table S14.1:** Correlations Between Factors from the Two‐Factor ESEM (Excluding Item 10 and Item 1) in Study 3.
**Table S15:** Factor Loadings from the Four‐Factor ESEM Applied to Study 3.
**Table S15.1:** Correlations Between Factors from the Four‐Factor ESEM in Study 3.
**Table S16:** Factor Loadings from the Four‐Factor ESEM (Excluding Item 1) Applied to Study 3.
**Table S16.1:** Correlations Between Factors from the Four‐Factor ESEM (Excluding Item 1) in Study 3.
**Table S17:** Factor Loadings from the Four‐Factor ESEM (Excluding Item 1 and Item 9) Applied to Study 3.
**Table S17.1:** Correlations Between Factors from the Four‐Factor ESEM (Excluding Item 1 and Item 9) in Study 3.
**Table S18:** Reliability of Scores Obtained from the RBQ‐3‐I Turkish Form for Study 3.

## Data Availability

The data that support the findings of this study are available from the corresponding author upon reasonable request.
