# Supplementary material for: A Factor‐Analytic Approach for Psychometric Properties of the Repetitive Behaviors Questionnaire‐3 (RBQ‐3) for Turkish Population
Source: Autism Res. 2026 Jul 15;19(8):e70311. doi: 10.1002/aur.70311 (PMC13471764; doi:10.1002/aur.70311)
Supplement: Supplementary file 1 [file AUR-19-0-s001.docx]

**Supplementary File**

A- **Results on the Adaptation of the RBQ-3-S into Turkish**

**Table S1**

*Factor Loadings from the Barrett et al. (2015) Two-Factor Model Applied to Study 1*

| **Items** | **Factor 1** | **Factor 2** |
| --- | --- | --- |
| Item 1 | 0.534^*^ | - |
| Item 2 | 0.786^*^ | - |
| Item 3 | 0.773^*^ | - |
| Item 4 | 0.743^*^ | - |
| Item 5 | 0.812^*^ | - |
| Item 6 | 0.828^*^ | - |
| Item 11 | - | 0.619^*^ |
| Item 12 | - | 0.563^*^ |
| Item 13 | - | 0.624^*^ |
| Item 14 | - | 0.697^*^ |
| Item 15 | - | 0.811^*^ |
| Item 16 | - | 0.720^*^ |
| Item 17 | - | 0.716^*^ |
| Item 19 | - | 0.614^*^ |

^*^:*p*<.05

**Table S1. 1.**

*Correlations Between Factors from the Barrett et al. (2015) Two-Factor Model in Study 1*

|  |  | **Factor 1** | **Factor 2** |
| --- | --- | --- | --- |
| **Factor 1** |  | 1.000 |  |
| **Factor 2** |  | 0.670^*^ | 1.000 |

^*^:*p*<.05

**Table S2.**

*Factor Loadings from the Barrett et al. (2018) Two-Factor Model Applied to Study 1*

| **Item** | **Factor 1** | **Factor 2** |
| --- | --- | --- |
| Item 1 | - | 0.477^*^ |
| Item 2 | 0.762^*^ | - |
| Item 3 | 0.776^*^ | - |
| Item 4 | 0.734^*^ | - |
| Item 5 | 0.807^*^ | - |
| Item 6 | 0.812^*^ | - |
| Item 7 | - | 0.635^*^ |
| Item 10 | 0.679^*^ | - |
| Item 11 | - | 0.617^*^ |
| Item 12 | - | 0.543^*^ |
| Item 13 | - | 0.590^*^ |
| Item 14 | - | 0.666^*^ |
| Item 15 | - | 0.787^*^ |
| Item 16 | - | 0.682^*^ |
| Item 18 | - | 0.628^*^ |
| Item 19 | - | 0.644^*^ |

^*^:*p*<.05

**Table S2.1.**

*Correlations Between Factors from the Barrett et al. (2018) Two-Factor Model in Study 1*

|  | **Factor 1** | **Factor 2** |
| --- | --- | --- |
| **Factor 1** | 1.000 |  |
| **Factor 2** | 0.771^*^ | 1.000 |

^*^:*p*<.05

**Table S3.**

*Factor Loadings from the Brett et al. (2024) Four-Factor Model Applied to Study 1*

| **Item** | **Factor 1** | **Factor 2** | **Factor 3** | **Factor 4** |
| --- | --- | --- | --- | --- |
| Item 2 | 0.764^*^ | - | - | - |
| Item 3 | 0.803^*^ | - | - | - |
| Item 4 | 0.749^*^ | - | - | - |
| Item 5 | 0.828^*^ | - | - | - |
| Item 6 | 0.817^*^ | - | - | - |
| Item 7 | - | 0.728^*^ | - | - |
| Item 8 | - | 0.828^*^ | - | - |
| Item 10 | - | 0.710^*^ | - | - |
| Item 11 | - | 0.664^*^ | - | - |
| Item 12 | - | 0.591^*^ | - | - |
| Item 13 | - | - | 0.658^*^ | - |
| Item 14 | - | - | 0.744^*^ | - |
| Item 15 | - | - | 0.871^*^ | - |
| Item 16 | - | - | 0.761^*^ | - |
| Item 17 | - | - | - | 0.820^*^ |
| Item 18 | - | - | - | 0.692^*^ |
| Item 19 | - | - | - | 0.715^*^ |

Note: ^*^:*p*<.05; The factor structure reported by Brett et al. (2024) in university students was used.

**Table S3. 1.**

*Correlations Between Factors from the Brett et al. (2024) Four-Factor Model in Study 1*

|  | **Factor 1** | **Factor 2** | **Factor 3** | **Factor 4** |
| --- | --- | --- | --- | --- |
| **Factor 1** | 1.000 | - | - | - |
| **Factor 2** | 0.827^*^ | 1.000 | - | - |
| **Factor 3** | 0.519^*^ | 0.625^*^ | 1.000 | - |
| **Factor 4** | 0.692^*^ | 0.699^*^ | 0.748^*^ | 1.000^*^ |

^*^:*p*<.05

**Table S4.**

*Factor Loadings from the Two-Factor ESEM Applied to Study 1*

| **Item** | **Factor 1** | **Factor 2** |
| --- | --- | --- |
| Item 1 | **0.418**^*^ | 0.129^*^ |
| Item 2 | **0.709**^*^ | 0.110^*^ |
| Item 3 | **0.872**^*^ | -0.079 |
| Item 4 | **0.660**^*^ | 0.120^*^ |
| Item 5 | **0.712**^*^ | 0.166^*^ |
| Item 6 | **0.779**^*^ | 0.061 |
| Item 7 | **0.678**^*^ | 0.090 |
| Item 8 | **0.645**^*^ | 0.247^*^ |
| Item 9 | **0.379**^*^ | 0.371^*^ |
| Item 10 | **0.541**^*^ | 0.280^*^ |
| Item 11 | **0.411**^*^ | 0.323^*^ |
| Item 12 | **0.383**^*^ | 0.262^*^ |
| Item 13 | -0.209^*^ | **0.825**^*^ |
| Item 14 | 0.057 | **0.694**^*^ |
| Item 15 | 0.079 | **0.784**^*^ |
| Item 16 | 0.040 | **0.733**^*^ |
| Item 17 | **0.420**^*^ | 0.409^*^ |
| Item 18 | 0.122^*^ | **0.586**^*^ |
| Item 19 | 0.116^*^ | **0.609**^*^ |

Note: ^*^:*p*<.05; Those shown in bold are higher factor loadings. Those shown in red are overlapping factor loadings.

**Table S4.1**

*Correlations Between Factors from the Two-Factor ESEM in Study 1*

|  | **Factor 1** | **Factor 2** |
| --- | --- | --- |
| **Factor 1** | 1.000 | - |
| **Factor 2** | 0.405^*^ | 1.000 |

^*^:*p*<.05

**Table S5.**

*Factor Loadings from the Two-Factor ESEM (Excluding Item 9) Applied to Study 1*

| **Item** | **Factor 1** | **Factor 2** |
| --- | --- | --- |
| Item 1 | **0.420**^*^ | 0.133^*^ |
| Item 2 | **0.709**^*^ | 0.112^*^ |
| Item 3 | **0.877**^*^ | -0.062 |
| Item 4 | **0.661**^*^ | 0.127^*^ |
| Item 5 | **0.709**^*^ | 0.167^*^ |
| Item 6 | **0.781**^*^ | 0.072 |
| Item 7 | **0.683**^*^ | 0.097 |
| Item 8 | **0.646**^*^ | 0.243^*^ |
| Item 10 | **0.522**^*^ | 0.251^*^ |
| Item 11 | **0.412**^*^ | 0.318^*^ |
| Item 12 | **0.386**^*^ | 0.262^*^ |
| Item 13 | -0.201^*^ | **0.822**^*^ |
| Item 14 | 0.060 | **0.688**^*^ |
| Item 15 | 0.089^*^ | **0.785**^*^ |
| Item 16 | 0.046 | **0.730**^*^ |
| Item 17 | **0.428**^*^ | 0.414^*^ |
| Item 18 | 0.131^*^ | **0.591**^*^ |
| Item 19 | 0.124^*^ | **0.611**^*^ |

Note: ^*^:*p*<.05; Those shown in bold are higher factor loadings. Those shown in red are overlapping factor loadings.

**Table S5.1.**

*Correlations Between Factors from the Two-Factor ESEM (Excluding Item 9) in Study 1*

|  | **Factor 1** | **Factor 2** |
| --- | --- | --- |
| **Factor 1** | 1.000 |  |
| **Factor 2** | 0.396^*^ | 1.000 |

^*^:*p*<.05

**Table S6.**

*Factor Loadings from the Two-Factor ESEM (Excluding Item 9 and Item 17) Applied to Study 1*

| **Item** | **Factor 1** | **Factor 2** |
| --- | --- | --- |
| Item 1 | **0.426**^*^ | 0.141^*^ |
| Item 2 | **0.707**^*^ | 0.111^*^ |
| Item 3 | **0.873**^*^ | -0.054 |
| Item 4 | **0.661**^*^ | 0.127^*^ |
| Item 5 | **0.714**^*^ | 0.178^*^ |
| Item 6 | **0.778**^*^ | 0.069 |
| Item 7 | **0.681**^*^ | 0.099 |
| Item 8 | **0.649**^*^ | 0.250^*^ |
| Item 10 | **0.524**^*^ | 0.253^*^ |
| Item 11 | **0.411**^*^ | 0.317^*^ |
| Item 12 | **0.389**^*^ | 0.266^*^ |
| Item 13 | -0.189^*^ | **0.817**^*^ |
| Item 14 | 0.068 | **0.684**^*^ |
| Item 15 | 0.103^*^ | **0.786**^*^ |
| Item 16 | 0.055 | **0.725**^*^ |
| Item 18 | 0.130^*^ | **0.580**^*^ |
| Item 19 | 0.126^*^ | **0.605**^*^ |

Note: ^*^:*p*<.05; Those shown in bold are higher factor loadings.

**Table S6.1.**

*Correlations Between Factors from the Two-Factor ESEM (Excluding Item 9 and Item 17) in Study 1*

|  | **Factor 1** | **Factor 2** |
| --- | --- | --- |
| **Factor 1** | 1.000 |  |
| **Factor 2** | 0.381^*^ | 1.000 |

^*^:*p*<.05

**Table S7.**

*Factor Loadings from the Four-Factor ESEM Applied to Study 1*

| **Item** | **Factor 1** | **Factor 2** | **Factor 3** | **Factor 4** |
| --- | --- | --- | --- | --- |
| Item 1 | **0.512**^*^ | 0.021 | 0.283^*^ | -0.143 |
| Item 2 | **0.670**^*^ | 0.155^*^ | 0.184^*^ | -0.028 |
| Item 3 | **0.624**^*^ | 0.245^*^ | -0.231^*^ | 0.269^*^ |
| Item 4 | **0.564**^*^ | 0.091 | 0.041 | 0.219^*^ |
| Item 5 | **0.521**^*^ | 0.259^*^ | 0.067 | 0.179^*^ |
| Item 6 | **0.761**^*^ | 0.014 | 0.051 | 0.174^*^ |
| Item 7 | 0.385^*^ | 0.369^*^ | -0.068 | 0.190^*^ |
| Item 8 | **0.247**^*^ | 0.567^*^ | 0.034 | 0.165^*^ |
| Item 9 | 0.021 | **0.621**^*^ | 0.276^*^ | -0.064 |
| Item 10 | 0.161 | **0.591**^*^ | 0.118^*^ | 0.056 |
| Item 11 | -0.049 | **0.616**^*^ | 0.047 | 0.205^*^ |
| Item 12 | 0.024 | **0.509**^*^ | 0.061 | 0.124 |
| Item 13 | -0.077 | -0.043 | **0.720**^*^ | 0.174^*^ |
| Item 14 | -0.016 | 0.237^*^ | **0.572**^*^ | 0.112^*^ |
| Item 15 | 0.167 | -0.023 | **0.646**^*^ | 0.273^*^ |
| Item 16 | -0.007 | 0.201^*^ | **0.602**^*^ | 0.139^*^ |
| Item 17 | 0.243 | 0.174^*^ | 0.146^*^ | **0.412**^*^ |
| Item 18 | -0.015 | -0.037 | 0.138^*^ | **0.733**^*^ |
| Item 19 | -0.047 | 0.004 | 0.148^*^ | **0.735**^*^ |

Note: *^*^:p*<.05; Those shown in bold are higher factor loadings. Those shown in red are overlapping factor loadings.

**Table S7.1.**

*Correlations Between Factors from the Four-Factor ESEM in Study 1*

|  | **Factor 1** | **Factor 2** | **Factor 3** | **Factor 4** |
| --- | --- | --- | --- | --- |
| **Factor 1** | 1.000 | - | - | - |
| **Factor 2** | 0.456^*^ | 1.000 | - | - |
| **Factor 3** | 0.161^*^ | 0.304^*^ | 1.000 | - |
| **Factor 4** | 0.366^*^ | 0.377^*^ | 0.407^*^ | 1.000 |

^*^:*p*<.05

**Table S8.**

*Factor Loadings from the Four-Factor ESEM (Excluding Item 7) Applied to Study 1*

| **Item** | **Factor 1** | **Factor 2** | **Factor 3** | **Factor 4** |
| --- | --- | --- | --- | --- |
| Item 1 | **0.503**^*^ | 0.027 | 0.285^*^ | -0.148 |
| Item 2 | **0.669**^*^ | 0.156^*^ | 0.180^*^ | -0.028 |
| Item 3 | **0.648**^*^ | 0.237^*^ | -0.220^*^ | 0.245^*^ |
| Item 4 | **0.568**^*^ | 0.099 | 0.031 | 0.219^*^ |
| Item 5 | **0.534**^*^ | 0.263^*^ | 0.059 | 0.172^*^ |
| Item 6 | **0.770**^*^ | 0.022 | 0.038 | 0.175^*^ |
| Item 8 | **0.268**^*^ | **0.521**^*^ | 0.072 | 0.136^*^ |
| Item 9 | 0.036 | **0.635**^*^ | 0.237^*^ | -0.053 |
| Item 10 | 0.181 | **0.589**^*^ | 0.096 | 0.059 |
| Item 11 | -0.033 | **0.636**^*^ | 0.007 | 0.214^*^ |
| Item 12 | 0.040 | **0.518**^*^ | 0.035 | 0.127 |
| Item 13 | -0.093 | -0.022 | **0.707**^*^ | 0.177^*^ |
| Item 14 | -0.026 | 0.247^*^ | **0.565**^*^ | 0.111^*^ |
| Item 15 | 0.159 | -0.027 | **0.664**^*^ | 0.261^*^ |
| Item 16 | -0.010 | 0.210^*^ | **0.599**^*^ | 0.132^*^ |
| Item 17 | 0.252 | 0.178^*^ | 0.140^*^ | **0.406**^*^ |
| Item 18 | 0.000 | -0.021 | 0.117^*^ | **0.740**^*^ |
| Item 19 | -0.027 | 0.013 | 0.147^*^ | **0.718**^*^ |

Note: ^*^:*p*<.05; Those shown in bold are higher factor loadings.

**Table S8.1.**

*Correlations Between Factors from the Four-Factor ESEM (Excluding Item 7) in Study 1*

|  | **Factor 1** | **Factor 2** | **Factor 3** | **Factor 4** |
| --- | --- | --- | --- | --- |
| **Factor 1** | 1.000 | - | - | - |
| **Factor 2** | 0.437^*^ | 1.000 | - | - |
| **Factor 3** | 0.176^*^ | 0.327^*^ | 1.000 | - |
| **Factor 4** | 0.346^*^ | 0.363^*^ | 0.415^*^ | 1.000 |

^*^:*p*<.05

**Table S9.**

*Reliability of Scores Obtained from the RBQ-3-S Turkish Form for Study 1*

| **Factor Analytic Method and Model** | **Factors** | **Cronbach’s Alpha (α)** | **McDonald’s Omega (ω)** |
| --- | --- | --- | --- |
| 2- Factor CFA (Barrett, 2015) | Factor 1 | .781 | .788 |
|  | Factor 2 | .809 | .811 |
|  | Total | .853 | .854 |
| 2-Factor CFA (Barrett, 2018) | Factor 1 | .794 | .804 |
|  | Factor 2 | .825 | .824 |
|  | Total | .875 | .876 |
| 4-Factor CFA (Brett et al., 2024) | Factor 1 | .782 | .791 |
|  | Factor 2 | .750 | .753 |
|  | Factor 3 | .793 | .795 |
|  | Factor 4 | .671 | .692 |
|  | Total | .884 | .886 |
| 2-Factor ESEM | Factor 1 | .865 | .869 |
|  | Factor 2 | .804 | .806 |
|  | Total | .890 | .892 |
| 2-Factor ESEM (Excluding Item 9) | Factor 1 | .858 | .862 |
|  | Factor 2 | .804 | .806 |
|  | Total | .885 | .886 |
| 2-Factor ESEM (Excluding Item 9 and Item 17) | Factor 1 | .804 | .806 |
|  | Factor 2 | .848 | .851 |
|  | Total | .875 | .877 |
| 4-Factor ESEM | Factor 1 | .825 | .830 |
|  | Factor 2 | .701 | .704 |
|  | Factor 3 | .793 | .795 |
|  | Factor 4 | .671 | .692 |
|  | Total | .890 | .892 |

**B-Results on the Adaptation of the RBQ-3-I into Turkish**

**Table S10.**

*Factor Loadings from the Lidstone et al. (2014)*, *Uljarević, Arnott, et al. (2017)* and *Uljarević,
Richdale, et al. (2017) Two-Factor Model Applied to Study 3*

| **Item** | **Factor 1** | **Factor 2** |
| --- | --- | --- |
| Item 2 | 0.786^*^ | - |
| Item 3 | 0.791^*^ | - |
| Item 4 | 0.804^*^ | - |
| Item 5 | 0.816^*^ | - |
| Item 6 | 0.753^*^ | - |
| Item 8 | 0.813^*^ | - |
| Item 10 | 0.742^*^ | - |
| Item 19 | 0.668^*^ | - |
| Item 7 | - | 0.847^*^ |
| Item 9 | - | 0.657^*^ |
| Item 12 | - | 0.634^*^ |
| Item 13 | - | 0.728^*^ |
| Item 14 | - | 0.705^*^ |
| Item 15 | - | 0.818^*^ |
| Item 16 | - | 0.707^*^ |
| Item 17 | - | 0.769^*^ |
| Item 18 | - | 0.662^*^ |

^*^:*p*<.05

**Table S10.1.**

*Correlations Between Factors from Lidstone et al. (2014)*, *Uljarević, Arnott, et al. (2017)* and *Uljarević, Richdale, et al. (2017) Two-Factor Model in Sample 3*

|  | **Factor 1** | **Factor 2** |
| --- | --- | --- |
| **Factor 1** | 1.000 |  |
| **Factor 2** | 0.842^*^ | 1.000 |

^*^:*p*<.05

**Table S11.**

*Factor Loadings from the Leekam (2007) Two-Factor Model Applied to Study 3*

| **Item** | **Factor 1** | **Factor 2** |
| --- | --- | --- |
| Item 1 | 0.690^*^ | - |
| Item 2 | 0.795^*^ | - |
| Item 3 | 0.795^*^ | - |
| Item 4 | 0.808^*^ | - |
| Item 5 | 0.819^*^ | - |
| Item 6 | 0.757^*^ | - |
| Item 8 | 0.768^*^ | - |
| Item 9 | 0.659^*^ | - |
| Item 10 | 0.736^*^ | - |
| Item 11 | - | 0.642^*^ |
| Item 13 | - | 0.760^*^ |
| Item 14 | - | 0.729^*^ |
| Item 15 | - | 0.850^*^ |
| Item 16 | - | 0.734^*^ |
| Item 17 | - | 0.785^*^ |
| Item 18 | - | 0.683^*^ |
| Item 19 | - | 0.683^*^ |

^*^:*p*<.05

**Table S11. 1.**

*Correlations Between Factors from the Leekam (2007) Two-Factor Model in Study 3*

|  | **Factor 1** | **Factor 2** |
| --- | --- | --- |
| **Factor 1** | 1.000 |  |
| **Factor 2** | 0.783^*^ | 1.000 |

^*^:*p*<.05

**Table S12.**

*Factor Loadings from the Two-Factor ESEM Applied to Study 3*

| **Item** | **Factor 1** | **Factor 2** |
| --- | --- | --- |
| Item 1 | **0.412**^*^ | 0.356^*^ |
| Item 2 | **0.748**^*^ | 0.121^*^ |
| Item 3 | **0.827**^*^ | 0.018 |
| Item 4 | **0.821**^*^ | 0.027 |
| Item 5 | **0.787**^*^ | 0.081^*^ |
| Item 6 | **0.803**^*^ | -0.007 |
| Item 7 | **0.650**^*^ | 0.273^*^ |
| Item 8 | **0.593**^*^ | 0.304^*^ |
| Item 9 | 0.269^*^ | **0.470**^*^ |
| Item 10 | **0.435**^*^ | 0.393^*^ |
| Item 11 | 0.185^*^ | **0.556**^*^ |
| Item 12 | 0.166^*^ | **0.582**^*^ |
| Item 13 | 0.048 | **0.756**^*^ |
| Item 14 | -0.052 | **0.819**^*^ |
| Item 15 | 0.132^*^ | **0.772**^*^ |
| Item 16 | -0.009 | **0.785**^*^ |
| Item 17 | 0.340^*^ | **0.526**^*^ |
| Item 18 | 0.263^*^ | **0.480**^*^ |
| Item 19 | 0.331^*^ | **0.416**^*^ |

Note: ^*^:*p*<.05; Those shown in bold are higher factor loadings. Those shown in red are overlapping factor loadings*.*

**Table S12. 1.**

*Correlations Between Factors from the Two-Factor ESEM in Study 3*

|  | **Factor 1** | **Factor 2** |
| --- | --- | --- |
| **Factor 1** | 1.000 |  |
| **Factor 2** | 0.493^*^ | 1.000 |

^*^:*p*<.05

**Table S13.**

*Factor Loadings from the Two-Factor ESEM (Excluding Item 10) Applied to Study 3*

| **Item** | **Factor 1** | **Factor 2** |
| --- | --- | --- |
| Item 1 | **0.419**^*^ | 0.361^*^ |
| Item 2 | **0.747**^*^ | 0.12^*^ |
| Item 3 | **0.826**^*^ | 0.026 |
| Item 4 | **0.820**^*^ | 0.036 |
| Item 5 | **0.785**^*^ | 0.088^*^ |
| Item 6 | **0.800**^*^ | -0.005 |
| Item 7 | **0.650**^*^ | 0.275^*^ |
| Item 8 | **0.593**^*^ | 0.304^*^ |
| Item 9 | 0.252^*^ | **0.459**^*^ |
| Item 11 | 0.187^*^ | **0.556**^*^ |
| Item 12 | 0.167^*^ | **0.581**^*^ |
| Item 13 | 0.057^*^ | **0.754**^*^ |
| Item 14 | -0.043 | **0.816**^*^ |
| Item 15 | 0.142^*^ | **0.770**^*^ |
| Item 16 | 0.002 | **0.782**^*^ |
| Item 17 | 0.343^*^ | **0.526**^*^ |
| Item 18 | 0.265^*^ | **0.479**^*^ |
| Item 19 | 0.329^*^ | **0.415**^*^ |

Note: ^*^:*p*<.05; Those shown in bold are higher factor loadings. Those shown in red are overlapping factor loadings.

**Table S13.1.**

*Correlations Between Factors from the Two-Factor ESEM in Study 3*

|  | **Factor 1** | **Factor 2** |
| --- | --- | --- |
| **Factor 1** | 1.000 |  |
| **Factor 2** | 0.484^*^ | 1.000 |

^*^:*p*<.05

**Table S14.**

*Factor Loadings from the Two-Factor ESEM (Excluding Item 10 and Item 1) Applied to Study 3*

| **Item** | **Factor 1** | **Factor 2** |
| --- | --- | --- |
| Item 2 | **0.740**^*^ | 0.116^*^ |
| Item 3 | **0.822**^*^ | 0.027 |
| Item 4 | **0.818**^*^ | 0.043 |
| Item 5 | **0.786**^*^ | 0.095^*^ |
| Item 6 | **0.799**^*^ | 0.000 |
| Item 7 | **0.655**^*^ | 0.279^*^ |
| Item 8 | **0.595**^*^ | 0.306^*^ |
| Item 9 | 0.261^*^ | **0.461**^*^ |
| Item 11 | 0.193^*^ | **0.554**^*^ |
| Item 12 | 0.174^*^ | **0.579**^*^ |
| Item 13 | 0.058^*^ | **0.750**^*^ |
| Item 14 | -0.039 | **0.812**^*^ |
| Item 15 | 0.149^*^ | **0.770**^*^ |
| Item 16 | 0.004 | **0.779**^*^ |
| Item 17 | 0.349^*^ | **0.527**^*^ |
| Item 18 | 0.269^*^ | **0.479**^*^ |
| Item 19 | 0.335^*^ | **0.415**^*^ |

Note: ^*^:p<.05; Those shown in bold are higher factor loadings.

**Table S14.1.**

*Correlations Between Factors from the Two-Factor ESEM (Excluding Item 10 and Item 1) in Study 3*

|  | **Factor 1** | **Factor 2** |
| --- | --- | --- |
| **Factor 1** | 1.000 |  |
| **Factor 2** | 0.473^*^ | 1.000 |

^*^:*p*<.05

**Table S15.**

*Factor Loadings from the Four-Factor ESEM Applied to Study 3*

| **Item** | **Factor 1** | **Factor 2** | **Factor 3** | **Factor 4** |
| --- | --- | --- | --- | --- |
| Item 1 | **0.428**^*^ | 0.243* | 0.414* | -0.158* |
| Item 2 | **0.578**^*^ | 0.361* | 0.036 | 0.048 |
| Item 3 | **0.694**^*^ | 0.193* | 0.045 | 0.071 |
| Item 4 | **0.710**^*^ | 0.004 | 0.104* | 0.190* |
| Item 5 | **0.662**^*^ | 0.002 | 0.122* | 0.243* |
| Item 6 | **0.648**^*^ | 0.064 | -0.001 | 0.228* |
| Item 7 | 0.332^*^ | **0.477*** | -0.037 | 0.279* |
| Item 8 | 0.286^*^ | **0.521*** | 0.004 | 0.210* |
| Item 9 | -0.065 | **0.333*** | 0.070 | 0.476* |
| Item 10 | 0.071^*^ | **0.340*** | -0.032 | 0.540* |
| Item 11 | -0.043 | **0.579*** | 0.219* | 0.082 |
| Item 12 | -0.093^*^ | **0.676**^*^ | 0.222^*^ | 0.038 |
| Item 13 | 0.090^*^ | 0.156^*^ | **0.692**^*^ | 0.017 |
| Item 14 | -0.070^*^ | 0.216^*^ | **0.649**^*^ | 0.102^*^ |
| Item 15 | 0.116^*^ | 0.024 | **0.658**^*^ | 0.263^*^ |
| Item 16 | -0.019 | 0.069 | **0.643**^*^ | 0.213^*^ |
| Item 17 | 0.125^*^ | 0.120^*^ | 0.267^*^ | **0.477**^*^ |
| Item 18 | 0.105^*^ | -0.026 | 0.293^*^ | **0.477**^*^ |
| Item 19 | 0.093^*^ | 0.046 | 0.144^*^ | **0.558**^*^ |

Note: ^*^:*p*<.05; Those shown in bold are higher factor loadings. Those shown in red are overlapping factor loadings*.*

**Table S15.1.**

*Correlations Between Factors from the Four-Factor ESEM in Study 3*

|  | **Factor 1** | **Factor 2** | **Factor 3** | **Factor 4** |
| --- | --- | --- | --- | --- |
| **Factor 1** | 1.000 | - | - | - |
| **Factor 2** | 0.394^*^ | 1.000 | - | - |
| **Factor 3** | 0.260^*^ | 0.435^*^ | 1.000 | - |
| **Factor 4** | 0.435^*^ | 0.490^*^ | 0.445^*^ | 1.000 |

^*^:*p<.05*

**Table S16.**

*Factor Loadings from the Four-Factor ESEM (Excluding Item 1) Applied to Study 3*

| **Item** | **Factor 1** | **Factor 2** | **Factor 3** | **Factor 4** |
| --- | --- | --- | --- | --- |
| Item 2 | **0.528**^*^ | 0.302^*^ | -0.031 | 0.183^*^ |
| Item 3 | **0.693**^*^ | 0.186^*^ | 0.031 | 0.077 |
| Item 4 | **0.763**^*^ | 0.034 | 0.155^*^ | 0.059 |
| Item 5 | **0.702**^*^ | 0.021 | 0.163^*^ | 0.144^*^ |
| Item 6 | **0.662**^*^ | 0.060 | 0.008 | 0.197^*^ |
| Item 7 | 0.333^*^ | **0.455**^*^ | -0.042 | 0.297^*^ |
| Item 8 | 0.276^*^ | **0.491**^*^ | -0.015 | 0.256^*^ |
| Item 9 | -0.021 | **0.370**^*^ | 0.109^*^ | 0.368^*^ |
| Item 10 | 0.091^*^ | **0.361**^*^ | 0.000 | 0.465^*^ |
| Item 11 | 0.003 | **0.598**^*^ | 0.244^*^ | 0.012 |
| Item 12 | -0.051 | **0.693**^*^ | 0.242^*^ | -0.018 |
| Item 13 | 0.131^*^ | 0.163^*^ | **0.696**^*^ | -0.017 |
| Item 14 | -0.033 | 0.227^*^ | **0.655**^*^ | 0.068 |
| Item 15 | 0.147^*^ | 0.025 | **0.661**^*^ | 0.243^*^ |
| Item 16 | -0.025 | 0.064 | **0.615**^*^ | 0.253^*^ |
| Item 17 | 0.099^*^ | 0.101^*^ | 0.236^*^ | **0.538**^*^ |
| Item 18 | 0.088^*^ | -0.024 | 0.270^*^ | **0.501**^*^ |
| Item 19 | 0.046 | 0.031 | 0.098^*^ | **0.642**^*^ |

Note: *^*^:p*<.05; Those shown in bold are higher factor loadings. Those shown in red are overlapping factor loadings.

**Table S16.1.**

*Correlations Between Factors from the Four-Factor ESEM (Excluding Item 1) in Study 3*

|  | **Factor 1** | **Factor 2** | **Factor 3** | **Factor 4** |
| --- | --- | --- | --- | --- |
| **Factor 1** | 1.000 | - | - | - |
| **Factor 2** | 0.397^*^ | 1.000 | - | - |
| **Factor 3** | 0.234^*^ | 0.416^*^ | 1.000 | - |
| **Factor 4** | 0.480^*^ | 0.501^*^ | 0.458^*^ | 1.000 |

^*^:*p*<.05

**Table S17.**

*Factor Loadings from the Four-Factor ESEM (Excluding Item 1 and Item 9) Applied to Study 3*

| **Item** | **Factor 1** | **Factor 2** | **Factor 3** | **Factor 4** |
| --- | --- | --- | --- | --- |
| Item 2 | **0.497**^*^ | 0.323^*^ | -0.046 | 0.204^*^ |
| Item 3 | **0.660**^*^ | 0.205^*^ | 0.005 | 0.113^*^ |
| Item 4 | **0.779**^*^ | 0.038 | 0.156^*^ | 0.045 |
| Item 5 | **0.701**^*^ | 0.034 | 0.156^*^ | 0.143^*^ |
| Item 6 | **0.657**^*^ | 0.075 | 0.008 | 0.189^*^ |
| Item 7 | 0.264^*^ | **0.479**^*^ | -0.085^*^ | 0.366^*^ |
| Item 8 | 0.222^*^ | **0.501**^*^ | -0.041 | 0.309^*^ |
| Item 10 | 0.161^*^ | **0.318**^*^ | 0.068^*^ | 0.360^*^ |
| Item 11 | 0.003 | **0.597**^*^ | 0.249^*^ | 0.003 |
| Item 12 | -0.084^*^ | **0.734**^*^ | 0.231^*^ | -0.017 |
| Item 13 | 0.150^*^ | 0.158^*^ | **0.694**^*^ | -0.012 |
| Item 14 | -0.014 | 0.221^*^ | **0.655**^*^ | 0.067 |
| Item 15 | 0.137^*^ | 0.037 | **0.636**^*^ | 0.278^*^ |
| Item 16 | -0.040 | 0.075^*^ | **0.593**^*^ | 0.287^*^ |
| Item 17 | 0.049 | 0.121^*^ | 0.202^*^ | **0.596**^*^ |
| Item 18 | 0.081^*^ | -0.001 | 0.263^*^ | **0.498**^*^ |
| Item 19 | 0.028 | 0.059 | 0.092^*^ | **0.635**^*^ |

Note: *^*^:p*<.05; Those shown in bold are higher factor loadings.

**Table S17.1.**

*Correlations Between Factors from the Four-Factor ESEM (Excluding Item 1 and Item 9) in Study 3*

|  | **Factor 1** | **Factor 2** | **Factor 3** | **Factor 4** |
| --- | --- | --- | --- | --- |
| **Factor 1** | 1.000 | - | - | - |
| **Factor 2** | 0.415*^*^* | 1.000 | - | - |
| **Factor 3** | 0.211*^*^* | 0.406*^*^* | 1.000 | - |
| **Factor 4** | 0.493*^*^* | 0.496*^*^* | 0.441*^*^* | 1.000 |

*^*^:p*<.05

**Table S18.**

*Reliability of Scores Obtained from the RBQ-3-I Turkish Form for Study 3*

| **Factor Analytic Method** | **Factors** | **Cronbach’s Alpha (α)** | **McDonald’s Omega (ω)** |
| --- | --- | --- | --- |
| 2-Factor CFA (Lidstone et al., (2004); Uljarević, Arnott, et al. (2017) and Uljarević, Richdale, et al. (2017)) | Factor 1 | .882 | .885 |
|  | Factor 2 | .886 | .887 |
|  | Total | .924 | .925 |
| 2-Factor CFA (Leekam et al., 2007) | Factor 1 | .887 | .888 |
|  | Factor 2 | .864 | .866 |
|  | Total | .921 | .922 |
| 2-Factor ESEM | Factor 1 | .901 | .902 |
|  | Factor 2 | .879 | .880 |
|  | Total | .929 | .930 |
| 2-Factor ESEM (Excluding Item 10) | Factor 1 | .897 | .898 |
|  | Factor 2 | .879 | .880 |
|  | Total | .925 | .926 |
| 2-Factor ESEM (Excluding Items 10 and Item 1) | Factor 1 | .896 | .897 |
|  | Factor 2 | .879 | .880 |
|  | Total | .922 | .923 |
| 4-Factor ESEM | Factor 1 | .873 | .875 |
|  | Factor 2 | .832 | .833 |
|  | Factor 3 | .844 | .844 |
|  | Factor 4 | .729 | .729 |
|  | Total | .929 | .930 |
| 4-Factor ESEM (Excluding Item 1) | Factor 1 | .874 | .875 |
|  | Factor 2 | .832 | .833 |
|  | Factor 3 | .844 | .844 |
|  | Factor 4 | .729 | .729 |
|  | Total | .926 | .927 |
